# Brazilian green propolis improves gut microbiota dysbiosis and protects against sarcopenic obesity

**DOI:** 10.1002/jcsm.13076

**Published:** 2022-09-26

**Authors:** Takuro Okamura, Masahide Hamaguchi, Ryo Bamba, Hanako Nakajima, Yuta Yoshimura, Tomonori Kimura, Yoshitaka Hashimoto, Saori Majima, Takafumi Senmaru, Emi Ushigome, Naoko Nakanishi, Mai Asano, Masahiro Yamazaki, Yuichiro Nishimoto, Takuji Yamada, Chizuru Fujikura, Takashi Asama, Nobuaki Okumura, Hiroshi Takakuwa, Ryoichi Sasano, Michiaki Fukui

**Affiliations:** ^1^ Department of Endocrinology and Metabolism, Graduate School of Medical Science Kyoto Prefectural University of Medicine Kyoto Japan; ^2^ Metabologenomics Inc. Tsuruoka Yamagata Japan; ^3^ Department of Life Science and Technology Tokyo Institute of Technology Tokyo Japan; ^4^ Institute for Bee Products and Health Science, R&D Department Yamada Bee Company, Inc Okayama Japan; ^5^ Agilent Technologies, Chromatography Mass Spectrometry Sales Department Life Science and Applied Markets Group Tokyo Japan; ^6^ AiSTI SCIENCE CO., Ltd Wakayama Japan

**Keywords:** Brazilian green propolis, Propolis, Sarcopenic obesity, Metabolite, Gut microbiota

## Abstract

**Introduction:**

Brazilian green propolis is an important honeybee product that is considered beneficial for health. Here, we examined the therapeutic potential of dietary supplementation with propolis against sarcopenic obesity using Db/Db mice.

**Methods:**

Db/m mice fed a normal diet alone and Db/Db mice fed normal diet alone, or supplemented with different amounts of propolis (0.08, 0.4 and 2%), were examined for effects on sarcopenic obesity.

**Results:**

Propolis improved the glucose tolerance (*P* < 0.001), increased the grip strength (*P* < 0.001) and the weight of soleus (*P* = 0.006) and plantaris muscles (*P* = 0.008). Moreover, propolis improved the non‐alcoholic fatty liver disease activity score (*P* < 0.001) and decreased the expression of genes related to inflammation, liver fibrosis and fatty acid metabolism. Propolis decreased the accumulation of saturated fatty acids in the liver and increased their excretion in faeces. With regard to the innate immunity, propolis decreased the ratio of M1 macrophages (*P* = 0.008) and Type 1 and 3 innate lymphoid cells to CD45‐positive cells (*P* < 0.001) and increased the ratio of M2 macrophages (*P* = 0.002) and ILC2s (*P* = 0.007) in the liver. Additionally, propolis decreased the expression of genes related to muscle atrophy and inflammation and the concentration of saturated fatty acids in the soleus muscle. 16S rRNA phylogenetic sequencing revealed that propolis increased the Bacteroidetes/Firmicutes ratio, and the abundance of Butyricicoccus and Acetivibrio genera. Gut microbiota related to the pentose phosphatase pathway and glycerolipid metabolism was more prevalent after the administration of propolis.

**Conclusions:**

This is the first study to demonstrate that propolis can improve sarcopenic obesity by improving dysbiosis due to overeating and provides new insights into diet–microbiota interactions during sarcopenic obesity.

## Introduction

The number of individuals with Type 2 diabetes is rapidly increasing worldwide. Complications associated with Type 2 diabetes reduce the quality of life of a person and heavily burden the medical economy.^1^ Thus, prevention of the progression of diabetic complications is an important task.

In recent years, muscle atrophy has been thought to be a complication of diabetes.^2^ It has become clear that muscle atrophy, that is, sarcopenia, and sarcopenic obesity are strongly associated with dietary patterns or metabolic disorders.^3^ In fact, we demonstrated the presence of muscle atrophy in patients with diabetes.^4^ Muscle atrophy is also a risk factor for decreased activities of daily living and mortality.^5^

The intestinal tract is inhabited by 500–1000 trillion gut microbes of 100–1000 trillion species, which collectively form the intestinal flora. Changes in gut microbiota, referred to as dysbiosis, have been reported to be associated with metabolic syndrome, such as obesity, diabetes and hypertension.^6^

Propolis, generally known as the ‘bee glue’, refers to the resinous substance accumulated by bees from different types of plants. Propolis and its extracts have numerous applications in the treatment of various diseases owing to their antiseptic, anti‐inflammatory, antioxidant, antibacterial, antimycotic, antifungal, antiulcer, anticancer and immunomodulatory properties.^7^ Brazilian green propolis has been reported to improve lipid metabolism, obesity^8^ and insulin resistance in humans^9^ and animals.^10^ However, there are currently no reports showing a relationship between Brazilian green propolis and sarcopenic obesity, although there are various reports showing an association between various bee products and sarcopenia.^11^

In this study, we investigated the effect of Brazilian green propolis on metabolic abnormalities through changes in the gut microbiota and immune responses. Furthermore, we investigated the effect of Brazilian green propolis on the gut microbiota.

## Materials and methods

### Mice

All experimental procedures were approved by the Committee for Animal Research, Kyoto Prefectural University of Medicine, Japan (approval number: M2020‐48).

#### Stage1

Seven‐week‐old male non‐diabetic heterozygous Db/m mice and diabetic homozygous Db/Db mice were purchased from Shimizu Laboratory Supplies (Kyoto, Japan). Starting 8 weeks of age, mice were fed either a standard diet (SD; 344.9 kcal/100 g, fat kcal 4.6%; CLEA Japan, Tokyo) or the same standard diet with propolis added (2% *w*/w in chow) for 8 weeks. The propolis powder, standardized to contain 8.0% artepillin C and 0.14% culifolin, derived from *Baccharis dracunculifolia*, was obtained from Yamada Bee Company, Inc. (Lot: LY3‐033, Okayama, Japan). Brazilian green propolis was homogenized with 10 times the volume of ethanol, stirred at room temperature for 12 h, and then the solution was filtered and evaporated until the solid content of the solution was 55% by weight. The total solids content was measured after evaporation by vacuuming.^12^ Initially, a preliminary experiment was conducted with two groups, Db/Db, Db/Db + 2%P, with *n* = 4 in each group. Because the main theme of this experiment was to improve sarcopenia obesity, the sample size was analysed using EZR with the relative grip strength as a guide; the mean difference between the two groups was 0.32, the mean standard deviation was 0.27, the significance level was 0.05, and the power was calculated at 80%, the required sample size was 12. Therefore, the sample size was set to 12. Twelve mice were assigned to the following four groups: (1) Db/m without propolis (Db/m), (2) Db/Db without propolis (Db/Db), (3) Db/Db with 0.08% propolis (Db/Db + 0.08%P), (4) Db/Db with 0.4% propolis (Db/Db + 0.4%P) and (5) Db/Db with 2% propolis (Db/Db + 2%P). Paired feeding was performed by supplying the same amount of feed among Db/Db mice.

#### Stage2

Faecal microbiota transplantation (FMT) has been broadly acknowledged as an approach to uncover the causal role of the gut microbiota in disease models related to the gut microbiota. To achieve better engraftment, depletion of recipient gut microbiota by antibiotics (ampicillin, neomycin, metronidazole: 1 g/L; vancomycin: 0.5 g/L, 200 μL/day by oral‐gastric gavage) was performed for 2 weeks from 6 to 8 weeks of age prior to FMT.^13^ After 3 days of recovery, FMT was performed twice weekly; 200–300 mg of fresh stool was collected from 16‐week‐old Db/Db mice and Db/Db + 2%P mice, respectively. The stool was homogenized in 5 mL of PBS, gravity sedimented for 2 min, and 200 μL of the supernatant was administered to each receiving mouse.^14^ Then, gut microbiota was extracted from the stools of 16‐week‐old Db/Db mice and Db/Db + 2%P mice, and FMT was performed. Between Db/Db mice treated with FMT of the stools of 16‐week‐old Db/Db mice (FMT (Db)) and Db/Db mice treated with FMT of the stools of 16‐week‐old Db/Db mice+2%P (FMT(P)), body weight changes, glucose tolerance and grip strength were evaluated, then sacrificed, and visceral fat and muscle mass were assessed.

**Figure 1:**
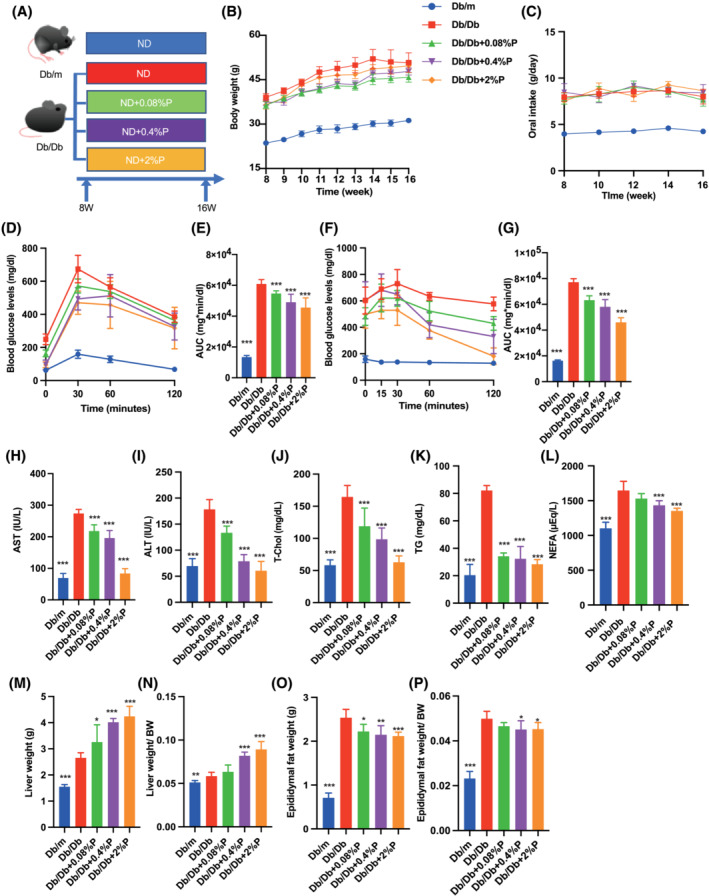
Administration of propolis improved obesity, glucose tolerance, hepatic enzymes, lipid metabolism and visceral fat obesity. (A) Administration of propolis (0.08, 0.4 and 2% per feed weight) started at 8 weeks of age. (B) Changes in the body weight (*n* = 12). (C) Oral intake (g/day) (*n* = 12). (D and E) Results of intraperitoneal glucose tolerance test (2 g/kg body weight) for 15‐week‐old mice and the area under the curve (AUC) analysis (*n* = 12). (F and G) Results of insulin tolerance test (0.75 U/kg body weight) for 15‐week‐old mice and the AUC analysis (*n* = 12). (H–L) Serum aspartate aminotransferase (AST), alanine aminotransferase (ALT), total cholesterol (T‐Chol), triglyceride (TG) and non‐esterified fatty acid (NEFA) levels (*n* = 12). (M and N) Absolute and relative weights of liver (*n* = 12). (O and P) Absolute and relative weights of epididymal fat (*n* = 12). Data are represented as the mean ± SD values. Data were analysed using one‐way ANOVA with Holm–Šídák's multiple‐comparisons test. *P < 0.05, **P < 0.01, ***P < 0.001.

At 16 weeks of age, after overnight fasting, all the mice were killed by administration of a combination of anaesthetics (0.3 mg/kg of medetomidine, 4.0 mg/kg of midazolam and 5.0 mg/kg of butorphanol) (*Figure* 1A).

### Glucose and insulin tolerance tests

The weights of mice were measured once a week after 14 h fasting overnight. Methods are described in detail in the Supporting Information.

### Biochemistry

Blood samples were taken from fasted mice, and aspartate aminotransferase (AST), alanine aminotransferase (ALT), total cholesterol (T‐Chol), triglyceride (TG) and non‐esterified fatty acid (NEFA) levels were measured. The biochemical examinations were performed at FUJIFILM Wako Pure 18 Chemical Corporation (Osaka, Japan).

### Liver histology

The liver tissue was obtained, fixed with 10% buffered formaldehyde and embedded in paraffin. Liver sections were prepared and stained with haematoxylin and eosin and Masson's trichrome stains. Images were captured with a BZ‐X710 fluorescence microscope (Keyence, Osaka, Japan). Additionally, to assess the severity of non‐alcoholic fatty acid disease (NAFLD), we determined the NAFLD activity score (NAS),^15^ which is a well‐known standard used for assessing the non‐alcoholic steatohepatitis (NASH) severity and measuring changes in NAFLD. Methods are described in detail in the Supporting Information.

### Gene expression analysis in murine liver soleus muscle, plantaris muscle and jejunum

The livers of 16‐h fasted mice were resected and immediately frozen in liquid nitrogen. Liver samples were homogenized in ice‐cold QIAzol Lysis reagent (Qiagen, Venlo, The Netherlands), and total RNA was isolated as described in the manufacturer's instructions. Total RNA (0.5 μg) was reverse‐transcribed using a High‐Capacity cDNA Reverse Transcription Kit (Applied Biosystems, Foster City, CA, USA) for first‐strand cDNA synthesis using an oligonucleotide dT primer and random hexamer priming according to the manufacturer's recommendations. Real‐time reverse transcription‐polymerase chain reaction (RT‐PCR) was used to quantify the mRNA expression levels of *Tnfa*, *Ccl2*, *Col1a*, *Fasn*, *Scd1* and *Elovl6* in the liver; of *Foxo1*, *Mstn*, *Hdac4*, *Fbxo32*, *Trim63*, *Il6* and *Tnfa* in the soleus and plantaris muscles; and of *Cd36* for the jejunum. The relative expression levels of target genes were normalized to threshold cycle (CT) values for *Gapdh* and quantified using the comparative threshold cycle 2^−ΔΔCT^ method. Signals from Db/m were assigned a relative value of 1.0. Six mice from each group were examined, and RT‐PCR was performed in triplicate for each sample. Methods are described in detail in the Supporting Information.

### Isolation of mononuclear cells from liver

The abdominal cavity of mice under deep anaesthesia was opened and a needle was inserted into the portal vein. Methods are described in detail in the Supporting Information.

### Tissue preparation and flow cytometry

Stained cells were analysed using FACS Canto II, and the data were analysed using the FlowJo Version 10 software (TreeStar, Ashland, OR, USA). Methods are described in detail in the Supporting Information.

### Measurement of grip strength

Grip strength was measured using a grip strength meter for mice (model DS2‐50N, IMADA Co., Ltd, Toyohashi, Japan) on another batch of 16‐week‐old mice. Six consecutive measurements per day were performed at 1‐min intervals. The investigators were blinded to the grouping of mice. Grip strength was normalized to the body weight.

### Measurement of free fatty acids in the sera, faeces and liver

The composition of palmitic acid (PA) in murine sera, faeces and liver was measured using gas chromatography–mass spectrometry (GC/MS) performed on Agilent 7890B/7000D (Agilent Technologies, Santa Clara, CA, USA).

### Measurement of short‐chain fatty acids and amino acid concentrations in faeces and serum samples

The short‐chain fatty acid (SCFA) composition of the murine rectal faeces and serum samples and the amino acid composition of the murine plantaris muscle were analysed using GC/MS performed on an Agilent 7890B/7000D System (Agilent Technologies). Methods are described in detail in the Supporting Information.

### Mouse skeletal muscle cell culture

C2C12 cells (a mouse myoblast cell line; KAC Co. Ltd., Kyoto, Japan) were plated in 24‐well plates and grown in Dulbecco's modified Eagle's medium (DMEM), supplemented with 20% foetal bovine serum (Day −2). The medium was changed every other day. When the cells reached 80% confluence, they were differentiated in DMEM, supplemented with 2% horse serum (differentiation medium) (Day 0). At 24 h after the change of medium, the cells were treated with ethanol (Ctrl), 200 μM PA or 200 μM PA and 100 μg/mL of propolis (PA + P), 10.6 μg/mL of artepillin C (PA + A) or 1.89 μg/mL of kaempferide (PA + K) (Day 1).^16^ In addition, from among the substances contained in propolis (Table 1), artepillin C and kaempferide were administered alone, and the volume was set to the amount contained in 100 μg of propolis (artepillin C: 10.6 μg/mL, kaempferide: 1.89 μg/mL). At 96 h (Day 5) post‐treatment, myotube cells were evaluated through various experiments.

**Table 1:** Compounds of Brazilian green propolis (Top 10)

No.	Compound	MW	Purity (%)	Content rate in propolis powder (%)
1	Artepillin C	300.2	99.2	10.60
2	Baccharin	364.2	99.6	3.00
3	Kaempferide	300.2	99.1	1.89
4	p‐Coumaric acid	163.9	>98	1.80
5	Drupanin	232.1	96.7	1.78
6	2,2‐Dimethylchromene‐6‐propenoic acid	230.2	99.4	1.19
7	Capillartemisin A	316.1	95	1.19
8	6‐Methoxykaempferide	330.2	97.7	1.08
9	Dihydrokaempferide	302.1	99.8	0.62
10	Culifolin	298.1	98	0.27

### Immunocytochemistry

C2C12 cells were cultured in eight‐well chamber slides, and immunocytochemistry was performed on Day 5. Methods are described in detail in the Supporting Information.

### Gene expression in C2C12 cells

Gene expression in C2C12 cells was analysed on Day 5. The medium was removed, and the cells were washed two times with cold PBS. Methods are described in detail in the Supporting Information.

### Murine macrophage cell culture and flow cytometry

Murine macrophage cells (RAW264.7; KAC Co., Ltd., Kyoto, Japan) were seeded in 24‐well plates and grown in DMEM, supplemented with 10% FBS. RAW264.7 cells were treated with ethanol, 200 μM PA or 200 μM PA and 100 μg/mL of propolis for 24 h. Thereafter, RAW264.7 cells were pretreated with phorbol myristic acid (PMA) at the indicated concentrations for 20 min prior to stimulation with 1 μM ionomycin for the release of cytokines.

Stained cells were analysed using FACS Canto II, and the data were analysed using the FlowJo Version 10 software (TreeStar). Methods are described in detail in the Supporting Information.

### ATP activity

C2C12 cells were cultured in 24‐well plates, and cellular ATP was extracted on Day 5 using an intracellular ATP assay kit (Toyo‐B‐Net, Tokyo, Japan) according to the manufacturer's instructions. The medium was removed; cells were washed two times with cold PBS and then treated with ATP extraction buffer (400 μL/well) at room temperature for 5 min. The lysate was then dispensed into 96‐well plates (on ice) in triplicate, and a luminescent reagent (100 μL/well) was added. ATP activity was quantitated as a measure of luminescence using an Orion L microplate luminometer (Berthold Detection Systems, Pforzheim, Germany).

### Measurement of cellular oxygen consumption rate in C2C12 cells

The oxygen consumption rate (OCR) of C2C12 cells was determined using a Seahorse Extracellular Flux Analyser XFp (Agilent Technologies). Methods are described in detail in the Supporting Information.

### 16S rRNA sequencing

Microbial DNA was extracted from frozen faecal samples using the QIAamp DNA Faeces Mini Kit (Qiagen, Venlo, The Netherlands), following the manufacturer's instructions. Methods are described in detail in the Supporting Information.

### Statistical analysis

The data were analysed using the JMP Version 13.0 software (SAS, Cary, NC, USA). A one‐way ANOVA with Holm–Šídák's multiple‐comparisons test was used to compare the results of different groups, and unpaired *t*‐test was used to compare the results of two groups. *P*‐values < 0.05 were accepted as statistically significant. Figures were generated using the GraphPad Prism software (Version 9.0; San Diego, CA, USA).

## Results

### Propolis improved glucose tolerance, hepatic enzymes, lipid metabolism and decreased visceral adipose tissue

Body weight of Db/Db mice was the highest, followed by that of Db/Db 0.08%P, Db/Db 0.4%P, Db/Db 2%P mice and Db/m mice (*Figure* 1B). No differences in food intake were observed between Db/Db mice because of pair feeding (*Figure* 1C). In addition, propolis improved glucose tolerance (*Figure* 1D and 1E) and insulin sensitivity in proportion to the dose (*Figure* 1F and 1G). Levels of the hepatic enzymes, AST and ALT, were increased in Db/Db mice, whereas they were decreased upon administration of propolis (*Figure* 1H and 1I). Similarly, T‐chol, TG, and NEFA levels, indicative of lipid metabolisms, were increased in Db/Db mice, whereas these were decreased upon administration of propolis (*Figure* 1J–L).

The absolute and relative weights of the liver increased upon administration of propolis (*Figure* 1M and 1N). In contrast, absolute and relative epididymal fat weight decreased upon administration of propolis (*Figure* 1O and 1P).

### Propolis improved intrahepatic fat accumulation and fibrosis and decreased the expression of genes related to inflammation, fibrosis and fatty acid metabolism

**Figure 2:**
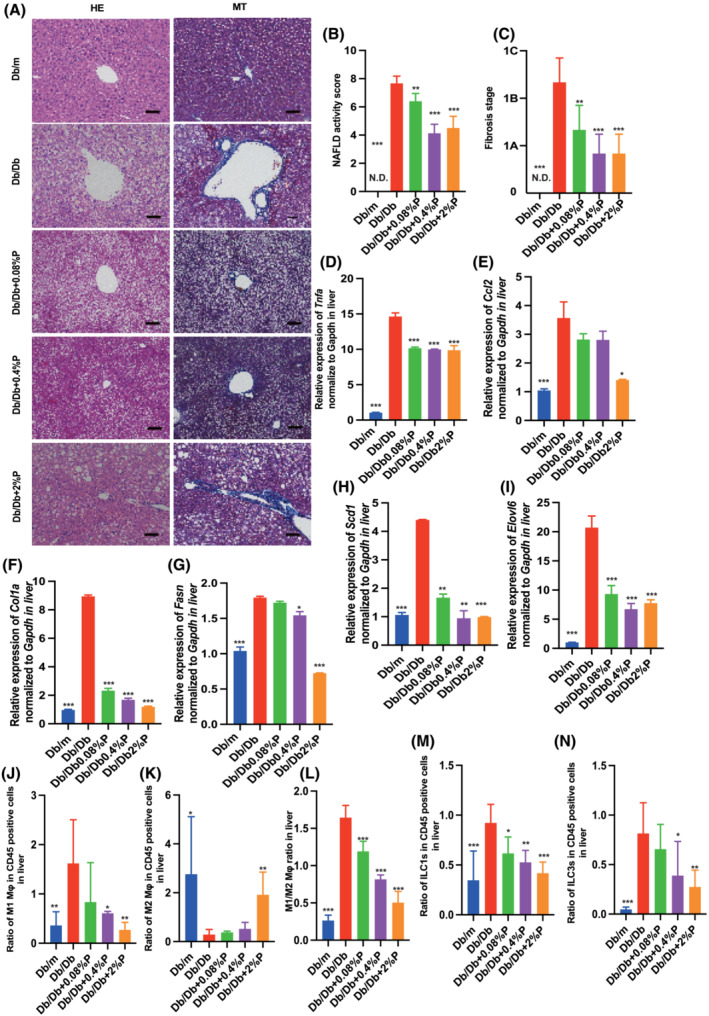
Administration of propolis improved fatty liver and decreased the expression of genes related to inflammation, liver fibrosis, and fatty acid metabolism, and decreased the ratio of M1 macrophages, ILC1, and ILC3, and increased the ratio of M2 macrophages and ILC2. (A) Representative images of haematoxylin and eosin‐stained and Masson trichrome‐stained liver sections. Liver tissues were collected at 16 weeks of age. The scale bar shows 100 μm. (B and C) Non‐alcoholic fatty liver disease (NAFLD) activity scores and the fibrosis stage of NAFLD activity score (*n* = 12). Relative *mRNA* expression of (D) *Tnfa*, (E) *Ccl2*, (F) C*ol1a*, (G) *Fasn*, (H) *Scd1* and (I) *Elovl6* in the liver normalized to the expression of *Gapdh* (*n* = 12). Ratio of (J) M1 macrophages to CD45‐positive cells, (K) M2 macrophages to CD45‐positive cells, (L) M1 to M2 macrophages, (M) ILC1s to CD45‐positive cells, (N) ILC3s to CD45‐positive cells in the liver (*n* = 12 in each case). Data are represented as the mean ± SD values. Data were analysed using one‐way ANOVA with Holm–Šídák's multiple‐comparisons test. *P < 0.05, **P < 0.01, ***P < 0.001. ND, not detected.

A representative image of the liver histology is shown in *Figure* 2A. Db/Db mice exhibited significant accumulation of fat (*Figure* 2B) and fibrosis (*Figure* 2C) in the liver, whereas propolis significantly improved these conditions. Moreover, administration of propolis decreased the expression of genes related to inflammation and fibrosis, such as *Tnf‐α*, *Ccl2* and *Col1a* (*Figure* 2D–F). The expression of genes related to fatty acid metabolism, such as *Fasn*, *Scd1* and *Elovl6*, in Db/Db Ctrl mice decreased after administration of propolis (*Figure* 2G–I).

### Propolis regulated inflammatory responses in innate immunity in liver

Next, we investigated the ratio of M1 (pro‐inflammatory) and M2 (anti‐inflammatory) macrophages in the liver. The ratio of M1 macrophages in Db/Db mice was significantly increased, compared with that in Db/m mice. In contrast, administration of propolis decreased the ratio of M1 macrophages (*Figure* 2J). Moreover, the ratio of M2 macrophages in Db/Db mice was significantly lower than that in Db/m mice, whereas that in Db/Db + 2%P mice was significantly increased (*Figure* 2K). The M1/M2 macrophage ratio in the liver of Db/Db mice was significantly increased, compared with that in Db/m mice, and administration of propolis decreased the ratio (*Figure* 2L).

The ratio of ILC1s in CD45‐positive cells of Db/Db mice was significantly higher than that in Db/m mice, and administration of propolis significantly decreased the ratio of ILC1s (*Figure* 2M). The ratio of ILC3s in CD45‐positive cells of Db/Db mice was also higher than that in Db/mice, and the administration of propolis decreased it, similar to that in the case of ILC1s (*Figure* 2N).

### Muscle weakness and loss of muscle mass observed in Db/Db mice were improved by propolis administration

**Figure 3:**
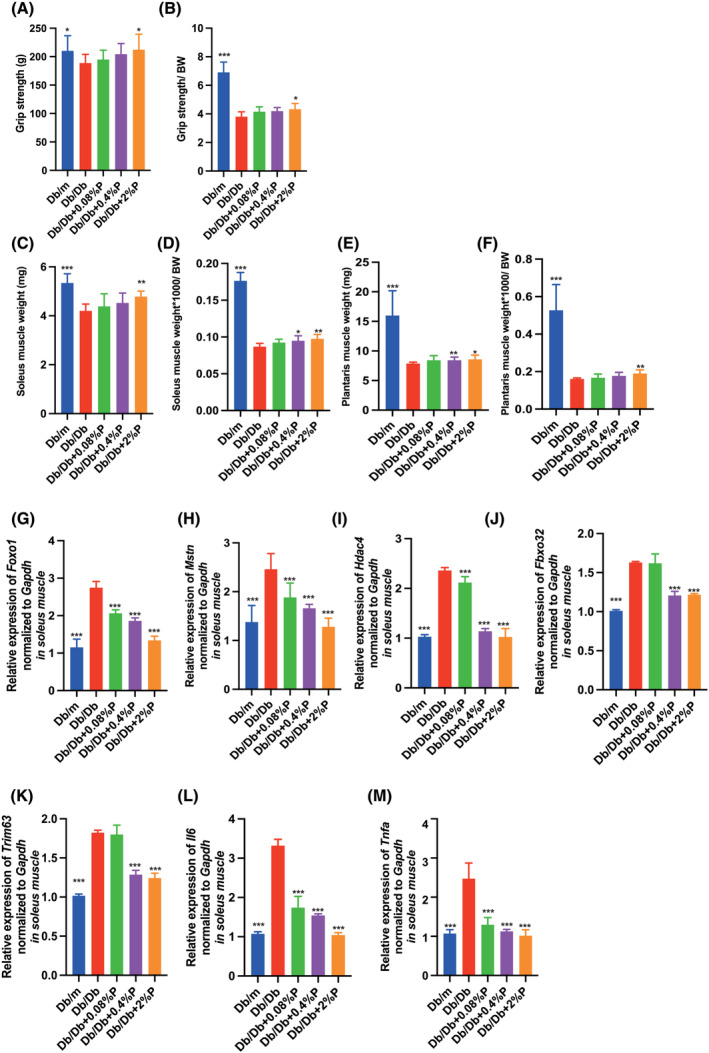
Administration of propolis increased the grip strength and muscle weight. (A and B) Absolute and relative grip strength, (C and D) absolute and relative soleus muscle weight, (E and F) absolute and relative plantaris muscle weight in 16‐week‐old mice (*n* = 12 in each case). Relative mRNA expression of (G) *Foxo1*, (H) *Mstn*, (I) *Hdac4*, (J) *Fbxo32*, (K) *Trim63*, (L) *Il6* and (I) *Tnfa* in the soleus muscle normalized to the expression of *Gapdh* (*n* = 12). Data are represented as the mean ± SD values. Data were analysed using one‐way ANOVA with Holm–Šídák's multiple‐comparisons test. *P < 0.05, **P < 0.01, ***P < 0.001.

Absolute and relative grip strength in Db/Db mice was significantly lower than that in Db/m mice, and administration of propolis increased the absolute and relative grip strength in a dose‐dependent manner, with a statistically significant difference observed in Db/Db + 2% P mice (*Figure* 3A and 3B) (A: *P* = 0.017, B: *P* = 0.002). The absolute and relative soleus and plantaris muscle weights in Db/Db mice were significantly lower than those in Db/m mice, and administration of propolis increased the absolute and relative muscle weight (*Figure* 3C–F) (compared with Db/Db + 2% P mice. C: *P* = 0.003, D: *P* = 0.006, E: *P* = 0.048, F: *P* = 0.008).

### Propolis decreased the expression of genes related to muscle atrophy and inflammation

In the RT‐PCR analysis, the expression of genes related to muscle atrophy and inflammation in the soleus muscle was determined. The expression of genes related to muscle atrophy, such as *Foxo1*, *Mstn*, *Hdac4* and *Fbxo32*, that is, MAFbx, and *Trim63*, that is, MuRF1, in Db/Db mice was significantly increased compared with that in Db/m mice, whereas the expression decreased following administration of propolis (*Figure* 3G–K). Similarly, the expression of genes related to inflammation, such as *Il6* and *Tnfa*, in Db/Db mice was significantly higher than that in Db/m mice, and the expression in Db/Db mice administered propolis was significantly lower than that in Db/Db mice (*Figure* 3L and 3M). The same results were obtained for the plantaris muscle (*Figure* S4A–G).

### Propolis increased the faecal excretion of PA and intestinal production of SCFAs

**Figure 4:**
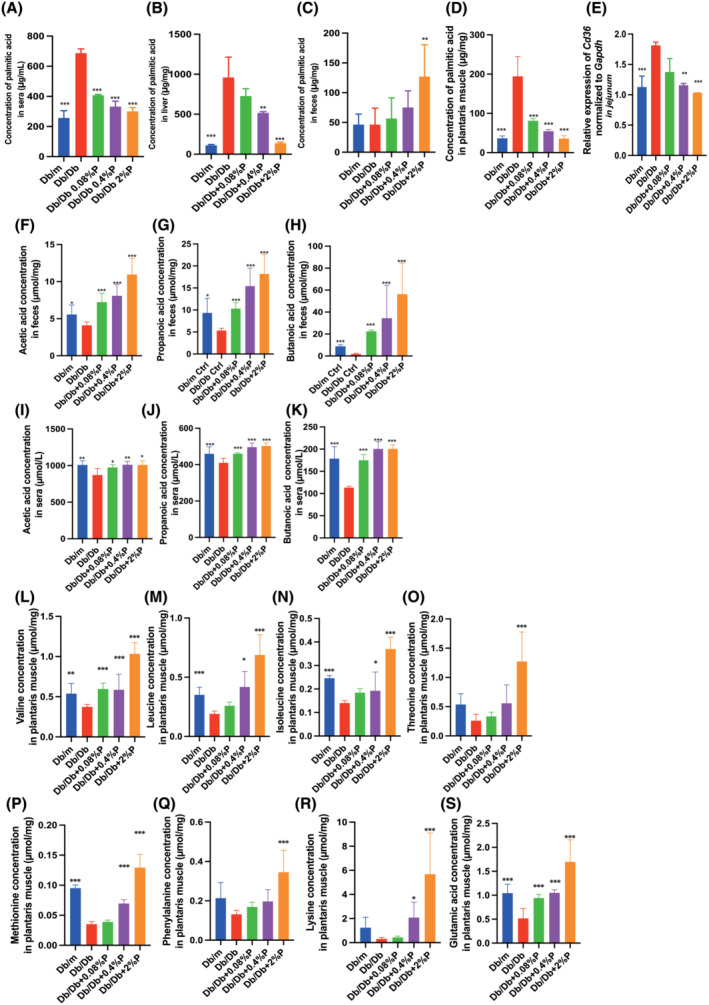
Administration of propolis decreased the Cd36 mRNA levels, which resulted in decreased concentration of saturated fatty acids in sera, liver and soleus muscle and increased concentration in faeces. The administration of propolis increased the concentration of short‐chain fatty acid in faeces and sera and the concentration of the amino acids related to muscle biosynthesis. The concentration of palmitic acid in (A) sera, (B) liver, (C) faeces and (D) soleus muscle (*n* = 12). (E) Relative mRNA expression of *Cd36* in the jejunum normalized to the expression of *GAPDH* (*n* = 12). The concentrations of (F) acetic acid, (G) propanoic acid and (H) butanoic acid in faeces (*n* = 12). The concentrations of (I) acetic acid, (J) propanoic acid and (K) butanoic acid in sera (*n* = 12). The concentrations of (L) valine, (M) leucine, (N) isoleucine, (O) threonine, (P) methionine, (Q) phenylalanine, (R) lysine and (S) glutamic acid in the plantaris muscle (*n* = 12). Data are represented as the mean ± SD values. Data were analysed using one‐way ANOVA with Holm–Šídák's multiple‐comparisons test. *P < 0.05, **P < 0.01, ***P < 0.001.

Next, we investigated the concentration of PA, a saturated fatty acid, in the sera, liver, faeces and plantaris muscle. The concentration of PA in the sera, liver and plantaris muscle of Db/Db mice was significantly higher than that in Db/m mice, whereas administration of propolis decreased the concentration in these samples (*Figure* 4A–D). On the contrary, the concentration of PA in the faeces of Db/Db mice was not different from that of Db/m mice; however, the concentration increased in a dose‐dependent manner, with a statistically significant difference in Db/Db + 2%P mice. The Cd36 mRNA levels in the jejunum of Db/Db mice were significantly higher than those in Db/m mice, whereas the administration of propolis decreased the expression (*Figure* 4E).

Furthermore, we investigated the concentration of SCFAs, such as acetic acid, propanoic acid, and butanoic acid, in faeces and sera. Acetic acid, propanoic acid and butyric acid concentrations in the faeces and sera of Db/Db mice were decreased compared with the respective concentrations in Db/m mice, whereas, interestingly, they were significantly increased upon propolis administration (*Figure* 4F–K).

### Propolis increased the concentrations of amino acids involved in protein synthesis in the plantaris muscle

We investigated the concentration of amino acids, such as valine, leucine, isoleucine, threonine, methionine, phenylalanine, lysine and glutamic acid, which are involved in protein synthesis in the plantaris muscle. The amino acid levels in Db/Db mice were significantly lower than those in Db/m mice, and administration of propolis increased the levels in a dose‐dependent manner (*Figure* 4L–S).

### Palmitic acid induced muscle atrophy and impaired mitochondria function

**Figure 5:**
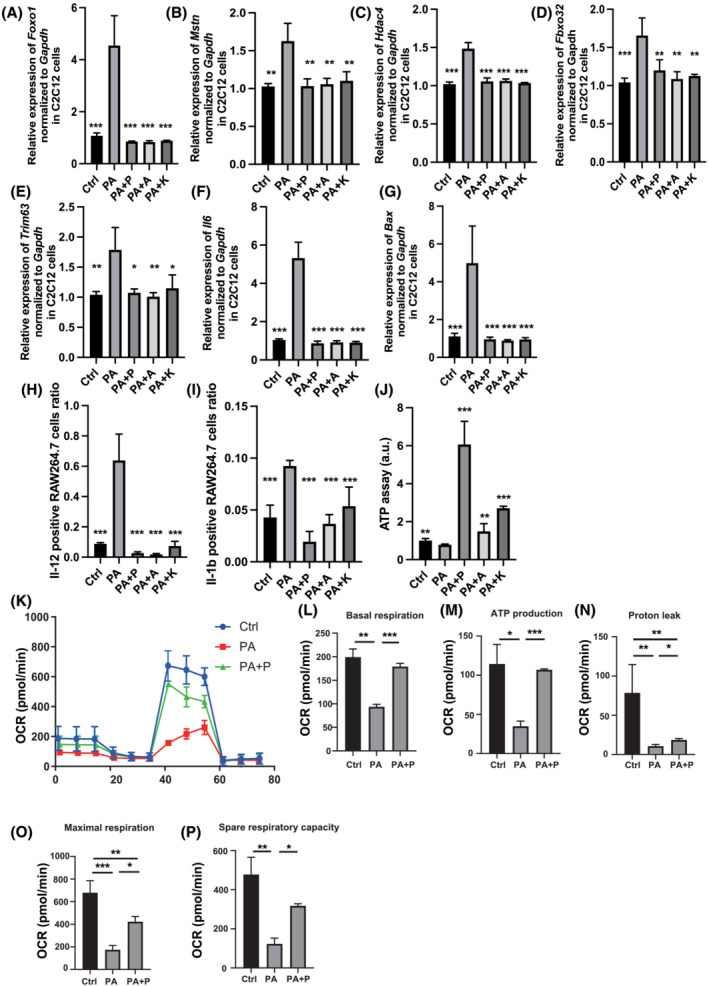
Administration of propolis protected against muscle atrophy and mitochondrial dysfunction induced by saturated fatty acid in C2C12 myotube cells. Relative mRNA expression of (A) *Foxo1*, (B) *Mstn*, (C) *Hdac4*, (D) *Fbxo32*, (E) *Trim63*, (F) *Il6* and (G) *Bax* in C2C12 myotube cells treated with ethanol (Ctrl), 200 μM palmitic acid (PA), 200 μM PA and 100 μg/mL propolis (PA + P) normalized to the expression of *Gapdh* (*n* = 6). (H) Ratio of Il‐12 + RAW264.7 cells in F4/80 + CD11b + cells treated with ethanol (Ctrl), 200 μM palmitic acid (PA), 200 μM PA and 100 μg/mL propolis (PA + P) (*n* = 6). (I) Ratio of Il‐1β + RAW264.7 cells in F4/80 + CD11b + cells treated with ethanol (Ctrl), 200 μM PA, 200 μM PA and 100 μg/mL propolis (PA + P) (*n* = 6). (J) ATP assay following treatment with ethanol (Ctrl), 200 μM palmitic acid (PA) or 200 μM PA and 100 μg/mL of propolis (PA + P), 10.6 μg/mL of artepillin C (PA + A) or 1.89 μg/mL of kaempferide (PA + K) (*n* = 6). (K) Raw data for oxygen consumption rate (OCR), (L) basal respiration, (M) ATP‐linked respiration (oligomycin‐sensitive OCR), (N) proton leak, (O) maximal mitochondrial respiration (FCCP‐stimulated OCR) and (P) spare respiratory capacity. OCRs were normalized to the total number of cells. Data are represented as the mean ± SD values. Data were analysed using one‐way ANOVA with Holm–Šídák's multiple‐comparisons test. *P < 0.05, **P < 0.01, ***P < 0.001.

The results presented in the previous section suggest that the increase in PA concentration in skeletal muscle is involved in muscle atrophy and the decrease upon administration of propolis might have a preventive effect on muscle atrophy. In view of this, we conducted experiments using C2C12 myotube cells. In RT‐PCR analyses, the relative expression of *Foxo1*, *Mstn*, *Hdac4*, *Fbxo32* and *Trim63*, which are related to muscle atrophy, *Il6*, which is related to inflammation, and *Bax*, which is related to apoptosis in C2C12 myotube cells increased upon administration of PA compared with that in the control group, whereas administration of propolis, artepillin C or kaempferide decreased the expression (*Figure* 5A–G).

Furthermore, experiments using the murine macrophage cell line, RAW264.7, were conducted, and the ratio of IL‐12‐ and IL‐1b‐positive cells was examined using flow cytometry. The administration of PA significantly increased the ratio of IL‐12‐positive and IL‐1b‐positive cells compared with that in the control group, whereas administration of propolis, artepillin C or kaempferide decreased the ratios (*Figure* 5H and 5I).

We hypothesized that propolis might have an inhibitory effect on mitochondrial dysfunction induced by PA and verified it using the ATP assay. As previously reported, PA significantly reduced the production of ATP in C2C12 myotube cells,^17^ whereas administration of propolis, artepillin C or kaempferide significantly increased the production (*Figure* 5J). Therefore, to further investigate mitochondrial function in detail, experiments were conducted using an extracellular flux analyser (*Figure* 5K). Basal respiration, ATP production, proton leak, maximal respiration and spare respiratory were significantly decreased upon administration of PA. In contrast, propolis improved the mitochondrial dysfunction induced by administration of PA (*Figure* 5L–P).

### Propolis improved dysbiosis in Db/Db mice

PCoA plots were constructed to compare the three groups of mice—Db/m mice, Db/Db mice and Db/Db + 2%P mice—which showed significant improvements in the grip strength and muscle mass (*Figure* S5A–C). The beta‐diversity determined using PERMANOVA tests (*Figure* S5D) showed notable differences between the Db/m and Db/Db mice (*P* = 0.001) and the Db/Db and Db/Db + 2%P mice (*P* = 0.006). In contrast, there were no significant differences between Db/m and Db/Db + 2%P mice (*P* = 0.124) (*Figure* S5D).

**Figure 6:**
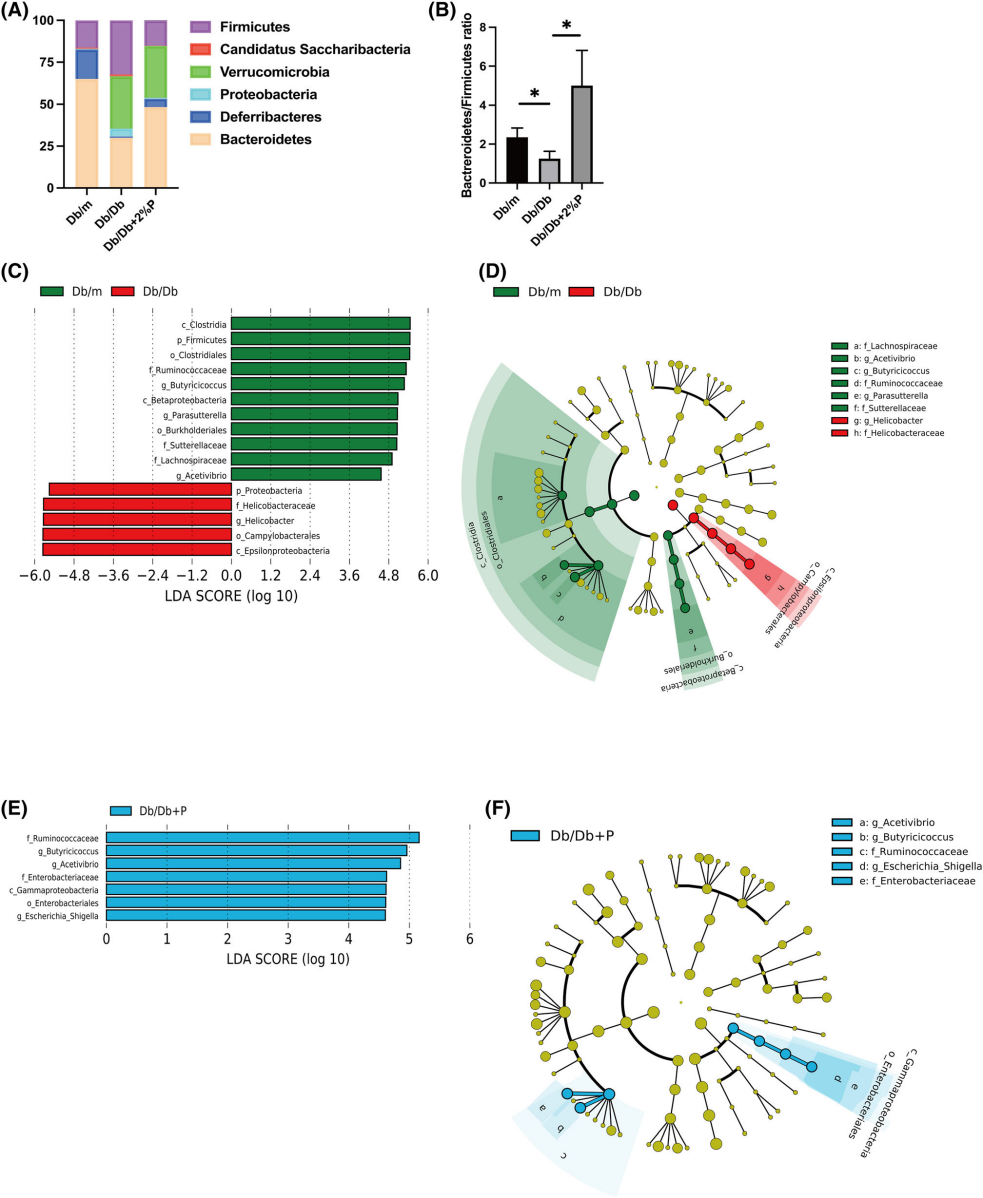
Components and functional profiles of the gut microbiota. (A) Relative abundance of gut microbiota at the phylum level (*n* = 3). (B) Ratio of the phylum Bacteroidetes in Firmicutes (*n* = 3). Data are the mean ± SD values. Data were analysed using one‐way ANOVA with Holm–Šídák's multiple‐comparisons test. *P < 0.05, as determined by one‐way ANOVA. The influence of genera on unique gut microbiota in Db/m mice, Db/Db mice and Db/Db + 2%P mice was assessed by LEfSe analysis (*n* = 3). (C and E) LDA scores of gut microbiota of Db/m and Db/Db + 2%P (green) and Db/Db (red) mice. (D and F) LEfSe was used to identify the taxa with the greatest differences in abundance between the gut microbiota of Db/m (Db/Db + 2%P) and Db/Db mice. Db/m mice (green); Db/Db mice (red); Db/Db + 2%P mice (blue). The brightness of each dot is proportional to the effect size. Only taxa with a significant LDA threshold value >2 are shown.

The most abundant phylum was Bacteroidetes in Db/m mice [mean relative abundance (SD), 65.1% (3.3%)] and Verrucomicrobia [31.3% (1.6%)] in Db/Db mice. Upon feeding propolis, the most abundant phylum was Bacteroidetes [48.3% (2.1%)]. Moreover, the ratio of Firmicutes in Db/Db mice decreased from 32.1 (3.4%) to 15.1% (8.6%) (*Figure* 6A). The Bacteroidetes/Firmicutes ratio in Db/Db mice was significantly lower than that in Db/m mice, whereas administration of propolis increased the ratio (*Figure* 6B).

We also used the LEfSe algorithm to identify the specific taxa that were variably distributed among the three groups. Five taxa were over‐represented (including the genus *Helicobacter* and the phylum Proteobacteria) and 11 were under‐represented (including the phyla Firmicutes and Proteobacteria and genus *Lactobacillus*) in the Db/Db mice compared with that in the Db/m mice (*Figure* 6C and 6D). Moreover, seven taxa were over‐represented in the Db/Db + 2%P mice compared with that in the Db/Db mice (including the family Ruminococcaceae, genus *Butyricicoccus* and genus *Acetivibrio*) (*Figure* 6E and 6F).

The Top 30 functional profiles for gut microbiota in Db/m and Db/Db mice, and Db/Db and Db/Db + 2%P mice were determined using the WAD algorithm, and functional profiles for gut microbiota were ranked from the top to the bottom, as shown in *Figure* S6A and S6B. Energy metabolism, amino acid‐related enzymes, valine, leucine and isoleucine biosynthesis, citrate cycle, sulfur metabolism, pyruvate metabolism, lipopolysaccharide biosynthesis proteins and alanine, aspartate, and glutamate metabolism in Db/Db mice were less prevalent than in Db/m mice. In contrast, starch and sucrose metabolism and amino sugar and nucleotide sugar metabolism in Db/Db mice were more prevalent than in Db/m mice. Carbon fixation pathways in prokaryotes, oxidative phosphorylation, porphyrin and chlorophyll metabolism, terpenoid backbone biosynthesis and photosynthesis proteins in Db/Db + 2%P mice were less prevalent, and pentose phosphatase pathway and glycerolipid metabolism, phenylalanine, galactose metabolism, tyrosine and tryptophan biosynthesis and glycerolipid metabolism were more prevalent than in Db/Db mice.

### Faecal transplantation

**Figure 7:**
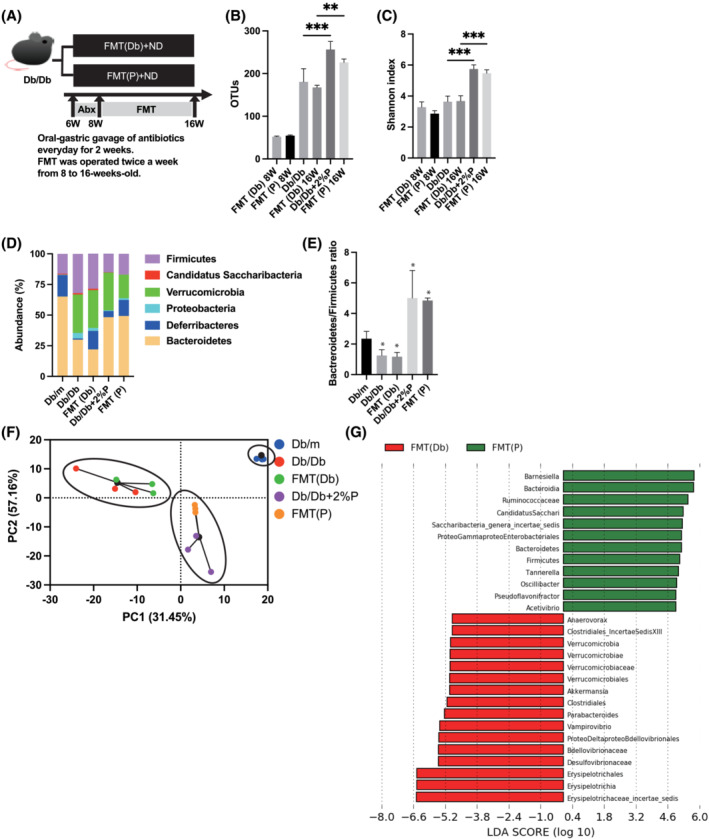
Faecal microbiota transplantation of Db/Db mice and Db/Db+2%P mice to recipient mice. (A) Oral‐gastric gavage of antibiotics was performed every day for 2 weeks. FMT was operated twice a week from 8 to 16 weeks old. (B) OTUs (*n* = 3). (C) Shannon‐index (*n* = 3). (D) Relative abundance of gut microbiota at the phylum level (*n* = 3). (E) Ratio of the phylum Bacteroidetes in Firmicutes (*n* = 3). (F) PCA and k‐means clustering for gut microbiota. (G) LDA scores of gut microbiota of FMT (Db) (green) and FMT(P) mice (red). Data are represented as the mean ± SD values. Data were analysed using one‐way ANOVA with Holm–Šídák's multiple‐comparisons test. Abx, antibiotics; FMT, faecal microbiota transplantation; LDA, linear discriminant analysis; ND, normal diet; PCA, principal component analysis.

To uncover the causal role of the gut microbiota and the metabolic improvement effect of propolis, FMT was performed (*Figure* 7A). Depletion of recipient gut microbiota by antibiotics was performed, and the number of operational taxonomic units (OTUs) in FMT (Db) and FMT (P) 8‐week‐old mice was lower than that in 16‐week‐old mice (*Figure* 7B). Shannon index, an indicator of diversity, of gut microbiota in FMT (Db) 16‐week‐old mice was lower than that in FMT (Db) 16‐week‐old mice (*Figure* 7C). The abundance of phylum was shown in *Figure* 7D. The most abundant phylum was Verrucomicrobia [mean relative abundance (SD), 30.9% (4.3%)] in FMT (Db) mice, same as Db/Db mice. Upon FMT of propolis, the most abundant phylum was Bacteroidetes [49.1% (3.9%)] same as Db/Db + 2%P mice. The Bacteroidetes/Firmicutes ratio of FMT (Db) mice was lower than that of FMT (P) mice (*Figure* 7E). Moreover, to investigate the effectiveness of FMT, principal component analyses were performed. The clustering result showed that Db/Db mice and FMT (Db) mice and Db/Db + 2%P mice and FMT(P) mice belonged to same cluster, respectively (*Figure* 7F). In addition, 12 taxa were over‐represented in the FMT(P) mice (including the genus *Barnesiella*, the class Bacteroidia, the family Ruminococcaceae and genus *Acetivibrio*), and 16 taxa were under‐represented compared with that in FMT (Db) mice (including the genera *Erysipelotrichaceae* and *Desulfovibrionaceae*).

**Figure 8:**
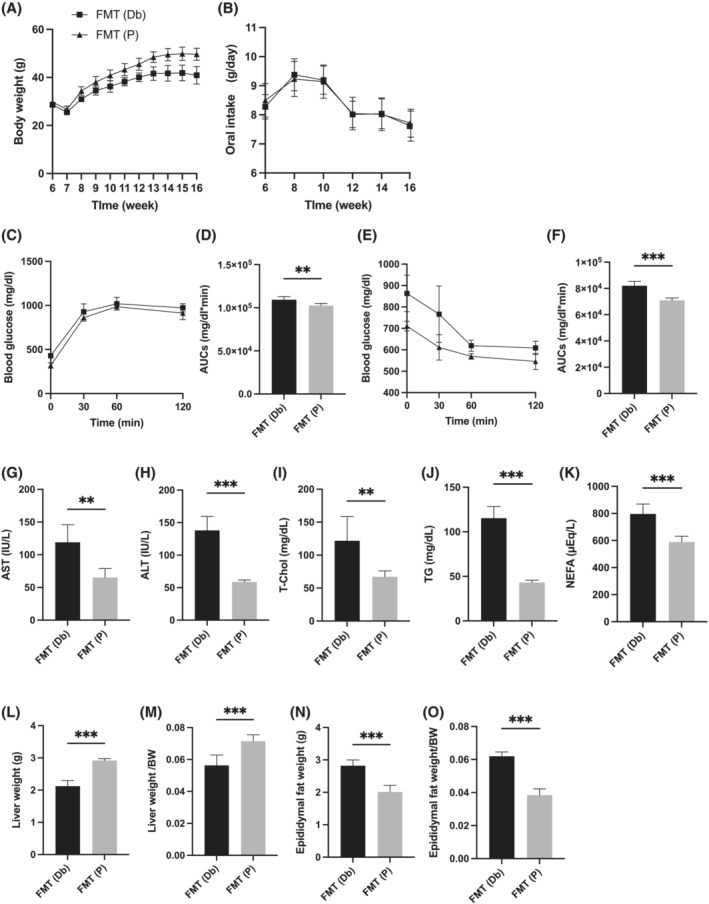
Faecal microbiota transplantation of Db/Db+2%P mice improved obesity, glucose tolerance, hepatic enzymes, lipid metabolism and visceral fat obesity. (A) Changes in the body weight (*n* = 6). (B) Oral intake (g/day) (*n* = 6). (C and D) Results of intraperitoneal glucose tolerance test (2 g/kg body weight) for 15‐week‐old mice and the area under the curve (AUC) analysis (*n* = 6). (E and F) Results of insulin tolerance test (0.75 U/kg body weight) for 15‐week‐old mice and the AUC analysis (*n* = 6). (G–K) Serum aspartate aminotransferase (AST), alanine aminotransferase (ALT), total cholesterol (T‐Chol), triglyceride (TG) and non‐esterified fatty acid (NEFA) levels (*n* = 6). (L and M) Absolute and relative weights of liver (*n* = 6). (N and O) Absolute and relative weights of epididymal fat (*n* = 6). Data are represented as the mean ± SD values. Data were analysed using unpaired *t*‐test. *P < 0.05, **P < 0.01, ***P < 0.001. FMT, faecal microbiota transplantation.

Body weight of FMT (Db) mice was lower than that of FMT (P) mice from 10 weeks of age (*Figure* 8A). Oral intake was shown in *Figure* 8B. No differences in food intake were observed between Db/Db mice because of pair feeding. In iPGTT and ITT, blood glucose levels of FMT (P) mice was lower than those of FMT (Db) mice (*Figure* 8C–F). The serum AST, ALT, T‐Chol, TG and NEFA levels in FMT (P) mice were lower than those in FMT (Db) mice (*Figure* 8G–K). Absolute and relative liver weight of FMT (P) mice were higher than those of FMT (Db) mice (*Figure* 8L and 8M). Absolute and relative epididymal fat weight of FMT (P) mice were lower than those of FMT (Db) mice (*Figure* 8N and 8O).

Next, liver histological analyses were performed (*Figure* S7A). NAS and fibrosis stage of FMT (P) mice were lower than that of FMT (Db) mice (*Figure* S7B and S7C). In addition, using flow cytometry analyses, macrophages and innate lymphoid cells were investigated in liver. The ratio of M1 macrophages in CD45‐positive cells in liver of FMT (P) mice were lower than that of FMT (Db), the ratio of M2 macrophages of FMT (P) mice were higher than that of FMT (Db) mice, and M1/M2 macrophages ratio in liver of FMT (P) mice was lower than that of FMT (Db) mice (*Figure* S7D–F). The ratio of ILC1s in CD45‐positive cells and the ratio of ILC3s in liver of FMT (P) mice were lower than that of FMT (Db) (*Figure* S7G and S7H).

Absolute and relative grip strength, soleus muscle weight and plantaris muscle weight of FMT (P) mice were higher than those of FMT (Db) mice (*Figure* S7I–N).

## Discussion

In the present study, administration of propolis improved dysbiosis, increased the excretion of saturated fatty acids in faeces and decreased the accumulation of saturated fatty acids in the liver and skeletal muscle, which resulted in improved NAFLD and sarcopenic obesity. However, this is the first study to show that propolis prevents sarcopenic obesity.

Propolis is a sticky resinous substance made by the bees by collecting plant resins, sap and sprouts and mixing them with their saliva and beeswax; it helps maintain a hygienic environment in the hive. Propolis has been used as a folk remedy since ancient times. Because it is made from plants, the constituent characteristics of propolis differ depending on the place of origin and plants of origin, that is, on the vegetation in the area where bees live.^18^ The Brazilian propolis used in this study is rich in artepillin C and kaempferide,^12^ which have been reported to have beneficial effects. Artepillin C shows anti‐inflammatory, antioxidant and antitumour effects,^19^ and kaempferide improves oxidative stress and inflammation.^20^ In this study, we found that administration of propolis and its components, artepillin C and kaempferide, protected against saturated fatty acid‐induced muscle atrophy. In experiments on C2C12 myotubular cells, administration of propolis and its components, artepillin C and kaempferide, not only suppressed muscle atrophy caused by saturated fatty acids but also, interestingly, improved mitochondrial dysfunction caused by these fatty acids. Mitochondria are not only the major producers of ATP but also major sources of reactive oxygen species (ROS) produced in electron transport chains as a by‐product of normal respiration.^21^ When ROS production increases chronically and exceeds the capacity of antioxidant defence mechanisms, ROS damage multiple cellular components, leading to oxidative stress.^22^ Recently, the production of ROS has emerged as an important link between excess free fatty acids, mitochondria and insulin resistance. Studies in rodents and obese individuals on high‐fat diets have shown increased mitochondrial ROS production in skeletal muscle in association with insulin resistance and no signs of mitochondrial respiratory failure.^23^ We have previously shown that saturated fatty acids released more IL‐12 when administered to RAW264.7 cells, a macrophage cell line, compared with the control group,^24^ whereas ILC1, which was reduced in the liver by propolis administration, released TNF‐α and IFN‐γ in response to IL‐12 and exhibited cytotoxic effects.^25^ Therefore, we hypothesized that the anti‐inflammatory effects of propolis and its constituents such as artepillin C and kaempferides in this study were partly due to the suppression of IL‐12 release from macrophages. Insulin resistance induced by saturated fatty acids, such as PA, has been reported to be associated with mitochondrial dysfunction in skeletal muscle cells,^26^ and PA has been shown to impair the activation of Akt. Akt activation is essential for the progression of mitochondrial respiratory stress signalling.

The relationship between gut microbiota and Type 2 diabetes has been studied in various ways. SCFAs produced by gut microbiota from indigestible nutrients have metabolic benefits for glucose control and anti‐inflammatory effects. This is because SCFAs inhibit the activation of nuclear factor‐kappa B by binding to FFA2 and FFA3, thereby, suppressing the release of tumour necrosis factor‐α (TNF‐α) and interleukin‐6 (IL‐6).^27^ Chronic inflammation increases insulin resistance in insulin‐sensitive organs, such as the liver and skeletal muscles, and leads to impaired glucose tolerance. In our study, propolis increased the production of SCFAs in faeces and decreased the expression of genes related to inflammation, such as *Tnfa* and *Il6*, in the liver and muscle. Xue et al.^28^ demonstrated that propolis increased the metabolic products of SCFAs and improved glucose tolerance in diabetic rats. Among the SCFAs, acetic acid and butyric acid, play an important role in maintaining the intestinal epithelial barrier function by increasing the expression of mucin from goblet cells and promoting the production of mucus. Impairment of the intestinal epithelial barrier allows for bacterial translocation of lipopolysaccharide produced by gram‐negative bacillus,^29^ resulting in a systemic microinflammatory state. Moreover, the proportion of phylum Proteobacteria, which is considered a potential indicator of gut dysbiosis,^30^ was significantly increased in the gut of Db/Db mice compared with that in Db/m mice, whereas the proportion of the family Ruminococcaceae, the genus *Butyricicoccus* and the genus *Acetivibrio* in the gut of Db/Db + 2%P mice was significantly increased compared with that in Db/Db mice. The family Ruminococcaceae has been reported to suppress obesity in humans.^31^ Moreover, the genus *Butyricicoccus* has been reported to produce butyric acid,^32^ and the genus *Acetivibrio* has been reported to ferment carbohydrates to produce acetic acid.^33^ Previous studies about propolis and gut microbiota using animals revealed that the abundant of phylum Firmicutes decreased^34^ and the abundance of the genus *Butyricicoccus increased* by the administration of propolis,^28^ which were similar to our study results. Furthermore, each of these studies on the effects of Brazilian propolis on dysbiosis, obesity and diabetes supports the efficacy of Brazilian propolis in this study. In addition, to show the causal role of the gut microbiota in improvement of metabolic abnormalities in Db/Db mice by propolis, FMT was performed. Then, the group transplanted with stools from propolis‐treated Db/Db mice not only exhibited the same gut microbiota as the propolis‐treated Db/Db mice but also significantly improved sarcopenia obesity as well as glucose tolerance and NAFLD compared with the group transplanted with stools from non‐propolis‐treated mice. Moreover, the family Ruminococcaceae and genus *Acetivibrio* also increased in FMT(P) mice, compared with FMT (Db) mice. Taken together, propolis administration improved dysbiosis and increased the abundance of gut microbiota that produces SCFAs.

In this study, administration of propolis increased the excretion of saturated fatty acids in faeces and decreased their accumulation in the liver and skeletal muscle. Saturated fatty acids in diet have received much attention for their ability to induce chronic low‐grade inflammation, widely recognized as a key link to obesity, Type 2 diabetes and cardiovascular disease.^35^ Additionally, intestinal levels of CD36 mRNA, which encodes one of the several putative plasma membrane long‐chain fatty acid transport proteins, were reported to be increased by feeding a high‐fat diet,^36^ suggesting that absorption of saturated fatty acids was increased by dysbiosis in the present study. Moreover, in previous studies, we have shown that saturated fatty acids cause inflammation in the liver by stimulating macrophages,^37^ and several previous studies have reported that saturated fatty acids can promote skeletal muscle atrophy.^38^ Overall, propolis treatment reduced the intestinal inflammation, CD36 expression and uptake of saturated fatty acids into the body by improving dysbiosis. This reduced the accumulation of fat in the liver and skeletal muscle, which might lead to improvements in fatty liver and sarcopenic obesity, respectively. Notably, propolis supplementation enlarged the livers of the mice without causing fatty liver. Possible explanation is that the inflammation and fibrosis caused by progress and the liver tends to atrophy in Db/Db mice. On the other hand, steatohepatitis is suppressed by propolis administration in Db/Db mice; however, fat deposition in the liver progresses, and the weight of the liver itself is larger than that in heterozygous mice. These results indicate that in Db/Db mice, administration of propolis did not reduce the weight of the liver but improves steatohepatitis.

In the functional profiling of gut microbiota, the pentose phosphatase pathway and glycerolipid metabolism were more prevalent following administration of propolis. The pentose phosphatase pathway is a major regulator of cellular reduction–oxidation homoeostasis and biosynthesis and modulates obesity‐induced inflammation and insulin sensitivity.^39^ An efficient ROS scavenging mechanism ensures protection against the detrimental effects of oxidative stress. Although several enzymes that remove ROS function in the cell, for their activity radical scavengers, such as thioredoxin and reduced glutathione, require NADPH, which is produced mainly through the pentose phosphate pathway. Moreover, glycerolipid metabolism has been reported to be involved in energy homoeostasis and thermogenesis,^40^ and the lipolytic segment of glycerolipid cycling produces free fatty acids, which in turn activate UCP‐1 in the brown adipose tissue to contribute to thermogenesis.^41^ In summary, the improvement of dysbiosis by propolis decreased oxidative stress and increased thermogenesis, which improved various metabolic abnormalities. Moreover, in the functional profiling of gut microbiota in Db/Db mice, it was found that the gut microbiota related to amino acid biosynthesis was decreased compared with that in Db/m mice, but it increased upon administration of propolis. In addition, the amino acid content related to skeletal muscle biosynthesis, which was decreased in Db/Db mice, was significantly increased upon administration of propolis, suggesting that propolis might have improved the skeletal muscle atrophy.

In conclusion, our findings indicate that propolis improves dysbiosis, resulting in a decrease in oxidative stress, an increase in thermogenesis, an increase in the excretion of saturated fatty acids into faeces due to the decrease in the expression of CD36 in the small intestine, an improvement in fatty liver and prevention of sarcopenia obesity by improving the mitochondrial function of skeletal muscle. Clinical studies are needed to assess the translational potential of our promising findings for the treatment of sarcopenic obesity.

## Conflict of interests

Takuro Okamura declares that he has no conflict of interest. Masahide Hamaguchi has received grants from Asahi Kasei Pharma, Nippon Boehringer Ingelheim Co., Ltd., Mitsubishi Tanabe Pharma Corporation, Daiichi Sankyo Co., Ltd., Sanofi K.K., Takeda Pharmaceutical Company Limited, Astellas Pharma Inc., Kyowa Kirin Co., Ltd., Sumitomo Dainippon Pharma Co., Ltd., Novo Nordisk Pharma Ltd. and Eli Lilly Japan K.K., outside the submitted work. Ryo Bamba declares that he has no conflict of interest. Hanako Nakajima declares that he has no conflict of interest. Yuta Yoshimura declares that he has no conflict of interest. Tomonori Kimura declares that he has no conflict of interest. Yoshitaka Hashimoto has received grants from Asahi Kasei Pharma, personal fees from Daiichi Sankyo Co., Ltd., personal fees from Mitsubishi Tanabe Pharma Corp., personal fees from Sanofi K.K. and personal fees from Novo Nordisk Pharma Ltd., outside the submitted work. Saori Majima declares that he has no conflict of interest. Takafumi Senmaru has received personal fees from Ono Pharma Co., Ltd., Mitsubishi Tanabe Pharma Co, Astellas Pharma Inc., Kyowa Hakko Kirin Co., Ltd., Sanofi K.K., MSD K.K., Kowa Pharma Co., Ltd., Taisho Toyama Pharma Co., Ltd., Takeda Pharma Co., Ltd., Kissei Pharma Co., Ltd., Novo Nordisk Pharma Ltd. and Eli Lilly Japan K.K. outside the submitted work. Emi Ushigome has received grants from the Japanese Study Group for Physiology and Management of Blood Pressure, the Astellas Foundation for Research on Metabolic Disorders (grant number: 4024). Donated Fund Laboratory of Diabetes therapeutics is an endowment department, supported with an unrestricted grant from Ono Pharmaceutical Co., Ltd., and received personal fees from AstraZeneca plc, Astellas Pharma Inc., Daiichi Sankyo Co., Ltd., Kyowa Hakko Kirin Company Ltd., Kowa Pharmaceutical Co., Ltd., MSD K.K., Mitsubishi Tanabe Pharma Corp., Novo Nordisk Pharma Ltd., Taisho Toyama Pharmaceutical Co., Ltd., Takeda Pharmaceutical Co., Ltd., Nippon Boehringer Ingelheim Co., Ltd. and Sumitomo Dainippon Pharma Co., Ltd., outside the submitted work. Naoko Nakanishi declares that he has no conflict of interest. Mai Asano Mai Asano received personal fees from Novo Nordisk Pharma Ltd., Abbott Japan Co., Ltd., AstraZeneca plc, Kowa Pharmaceutical Co., Ltd., Ono Pharmaceutical Co., Ltd., and Takeda Pharmaceutical Co., Ltds., outside the submitted work. Masahiro Yamazaki reports personal fees from MSD K.K., Sumitomo Dainippon Pharma Co., Ltd., Kowa Company, Limited, AstraZeneca PLC, Takeda Pharmaceutical Company Limited, Kyowa Hakko Kirin Co., Ltd., Daiichi Sankyo Co., Ltd., Kowa Pharmaceutical Co., Ltd., and Ono Pharma Co., Ltd., outside the submitted work. Yuichiro Nishimoto was employed by Metabologenomics Inc. Takuji Yamada was employed by Metabologenomics Inc. Chizuru Fujikura was employed by Yamada Bee Company, Inc. Takashi Asama was employed by Yamada Bee Company, Inc. Nobuaki Okumura was employed by Yamada Bee Company, Inc. Hiroshi Takakuwa was employed by Agilent Technologies. Ryoichi Sasano declares that he has no conflict of interest. Michiaki Fukui has received grants from Nippon Boehringer Ingelheim Co., Ltd., Kissei Pharma Co., Ltd., Mitsubishi Tanabe Pharma Co, Daiichi Sankyo Co., Ltd., Sanofi K.K., Takeda Pharma Co., Ltd., Astellas Pharma Inc., MSD K.K., Kyowa Hakko Kirin Co., Ltd., Sumitomo Dainippon Pharma Co., Ltd., Kowa Pharmaceutical Co., Ltd., Novo Nordisk Pharma Ltd., Ono Pharma Co., Ltd., Sanwa Kagaku Kenkyusho Co., Ltd. Eli Lilly Japan K.K., Taisho Pharma Co., Ltd., Terumo Co., Teijin Pharma Ltd., Nippon Chemiphar Co., Ltd., and Johnson & Johnson K.K. Medical Co., Abbott Japan Co., Ltd., and received personal fees from Nippon Boehringer Ingelheim Co., Ltd., Kissei Pharma Co., Ltd., Mitsubishi Tanabe Pharma Corp., Daiichi Sankyo Co., Ltd., Sanofi K.K., Takeda Pharma Co., Ltd., Astellas Pharma Inc., MSD K.K., Kyowa Kirin Co., Ltd., Sumitomo Dainippon Pharma Co., Ltd., Kowa Pharma Co., Ltd., Novo Nordisk Pharma Ltd., Ono Pharma Co., Ltd., Sanwa Kagaku Kenkyusho Co., Ltd., Eli Lilly Japan K.K., Taisho Pharma Co., Ltd., Bayer Yakuhin, Ltd., AstraZeneca K.K., Mochida Pharma Co., Ltd., Abbott Japan Co., Ltd., Medtronic Japan Co., Ltd., Arkley Inc., Teijin Pharma Ltd. and Nipro Cor., outside the submitted work.

## Funding

This research received funding from Yamada Bee Company, Inc.

## Supporting information


**Figure S1.** Strategy for innate lymphoid cells (ILCs). Representative flow cytometry plots of liver CD45 + Live & Dead‐ Lin‐ CD127 + RORg‐ GATA‐3‐ T‐bet+ ILC1s, CD45 + Live & Dead‐ Lin‐ CD127 + RORg‐ GATA‐3 + ILC2s and CD45 + Live & Dead‐ Lin‐ CD127 + RORg+ GATA‐3‐ ILC3s in each group at 16‐weeks of age.
**Figure S2**. Strategy for macrophages. Representative flow cytometry plots of liver CD45 + F4/80 + CD206‐ CD11c + M1 macrophages and CD45 + F4/80 + CD206‐ CD11c + M2 macrophages in each group at 16 weeks of age.
**Figure S3**. Strategy for macrophages. Representative flow cytometry plots of RAW264.7 cells F4/80 + CD11b + IL12 cells and F4/80 + CD11b + IL1β cells.
**Figure S4**. The gene expressions in plantaris muscle. Relative expression of mRNA (A) Foxo1, (B) Mstn, (C) Hdac4, (D) Fbxo32, (E) Trim63, (F) Il6, and (G) Tnfa of indicated genes in soleus muscle normalized to Gapdh (*n* = 12). Data have been represented in terms of mean ± SD values. ***p < 0.001 by one‐way ANOVA.
**Figure S5**. PCoA plots of gut microbiota of Db/m, Db/Db, and Eb/DB + 2%P mice. Weighted PCoA plots were shown (*n* = 3). (A) PC1 vs PC2, (B) PC2 vs PC3, and (C) PC1 vs PC3. (D) Weighted beta‐diversity with PERMANOVA test were shown (n = 3). PCoA: Principal coordinate analysis, PERMANOVA: Permutational multivariat analysis of variance.
**Figure S6**. The functional profiles for gut microbiota. (A and B) Change in metabolism profiles for the gut microbiota. The degree of impairment in the gut microbial metabolism profile for Db/m mice, Db/Db mice, and Db/Db + 2%P mice was assessed by the weighted average differences method, and the degree was ranked from top to bottom. The top 20 metabolism profiles for gut microbiota are shown, and the difference of these gut microbiota profiles was evaluated by unpaired t‐tests. Histograms show the mean proportion of the relative abundance of metabolism profiles of gut microbiota (mean + standard deviation) and p‐ value by unpaired t‐test is shown.
**Figure S7**. Fecal microbiota transplantation of Db/Db + 2%P mice improved fatty liver, decreased the expression of genes related to inflammation, liver fibrosis, and fatty acid metabolism, and decreased the ratio of M1 macrophages, ILC1, and ILC3, and increased the ratio of M2 macrophages and ILC2, and increased the muscle strength and mass. (A) Representative images of hematoxylin & eosin‐ and Masson trichrome‐stained liver sections. Liver tissues were collected at 16‐weeks of age. The scale bar shows 100 μm. (B and C) Nonalcoholic fatty liver disease (NAFLD) activity scores and the fibrosis stage of NAFLD activity score (*n* = 6). Ratio of (D) M1 macrophages to CD45‐positive cells, (E) M2 macrophages to CD45‐positive cells, (F) M1 to M2 macrophages, (G) ILC1s to CD45‐positive cells, (H) ILC3s to CD45‐positive cells in the liver (*n* = 6 in each case). (I and J) Absolute and relative grip strength, (K and L) absolute and relative soleus muscle weight, (M and N) absolute and relative plantaris muscle weight in 16‐weeks‐old mice (*n* = 12 in each case). Data are represented as the mean ± SD values. Data were analyzed using unpaired t‐test. *p < 0.05, **p < 0.01, ***p < 0.001. FMT, fecal microbiota transplantation.

**Data S1.** Supporting Information
