# Supplementary material for: Brazilian green propolis improves gut microbiota dysbiosis and protects against sarcopenic obesity
Source: J Cachexia Sarcopenia Muscle. 2022 Sep 26;13(6):3028–47. doi: 10.1002/jcsm.13076 (PMC9745478; doi:10.1002/jcsm.13076)
Supplement: Supplementary file 1 — Figure S1. Strategy for innate lymphoid cells (ILCs). Representative flow cytometry plots of liver CD45 + Live & Dead‐ Lin‐ CD127 + RORg‐ GATA‐3‐ T‐bet+ ILC1s, CD45 + Live & Dead‐ Lin‐ CD127 + RORg‐ GATA‐3 + ILC2s and CD45 + Live & Dead‐ Lin‐ CD127 + RORg+ GATA‐3‐ ILC3s in each group at 16‐weeks of age. Figure S2. Strategy for macrophages. Representative flow cytometry plots of liver CD45 + F4/80 + CD206‐ CD11c + M1 macrophages and CD45 + F4/80 + CD206‐ CD11c + M2 macrophages in each group at 16 weeks of age. Figure S3. Strategy for macrophages. Representative flow cytometry plots of RAW264.7 cells F4/80 + CD11b + IL12 cells and F4/80 + CD11b + IL1β cells. Figure S4. The gene expressions in plantaris muscle. Relative expression of mRNA (A) Foxo1, (B) Mstn, (C) Hdac4, (D) Fbxo32, (E) Trim63, (F) Il6, and (G) Tnfa of indicated genes in soleus muscle normalized to Gapdh (n = 12). Data have been represented in terms of mean ± SD values. ***p < 0.001 by one‐way ANOVA. Figure S5. PCoA plots of gut microbiota of Db/m, Db/Db, and Eb/DB + 2%P mice. Weighted PCoA plots were shown (n = 3). (A) PC1 vs PC2, (B) PC2 vs PC3, and (C) PC1 vs PC3. (D) Weighted beta‐diversity with PERMANOVA test were shown (n = 3). PCoA: Principal coordinate analysis, PERMANOVA: Permutational multivariat analysis of variance. Figure S6. The functional profiles for gut microbiota. (A and B) Change in metabolism profiles for the gut microbiota. The degree of impairment in the gut microbial metabolism profile for Db/m mice, Db/Db mice, and Db/Db + 2%P mice was assessed by the weighted average differences method, and the degree was ranked from top to bottom. The top 20 metabolism profiles for gut microbiota are shown, and the difference of these gut microbiota profiles was evaluated by unpaired t‐tests. Histograms show the mean proportion of the relative abundance of metabolism profiles of gut microbiota (mean + standard deviation) and p‐ value by unpaired t‐test is shown. Figure S7. Fecal microbiota tran [file JCSM-13-3028-s001.pdf]

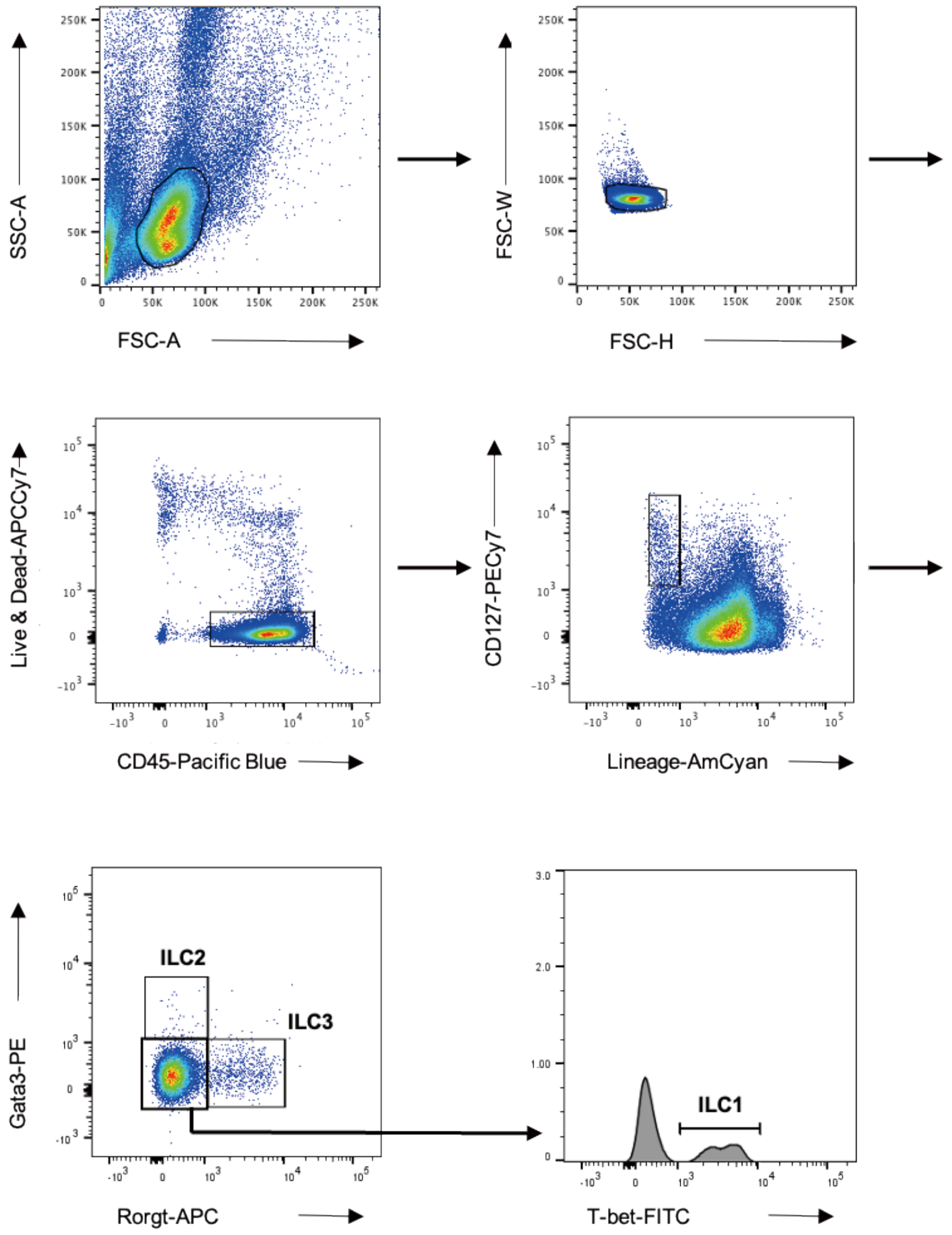

Supplementary Figure 1. Strategy for innate lymphoid cells (ILCs)

Representative flow cytometry plots of liver CD45+ Live & Dead- Lin- CD127+ RORg- GATA-3- T-bet+ ILC1s, CD45+ Live & Dead- Lin- CD127+ RORg- GATA-3+ ILC2s and CD45+ Live & Dead- Lin- CD127+ RORg+ GATA-3- ILC3s in each group at 16-weeks of age.

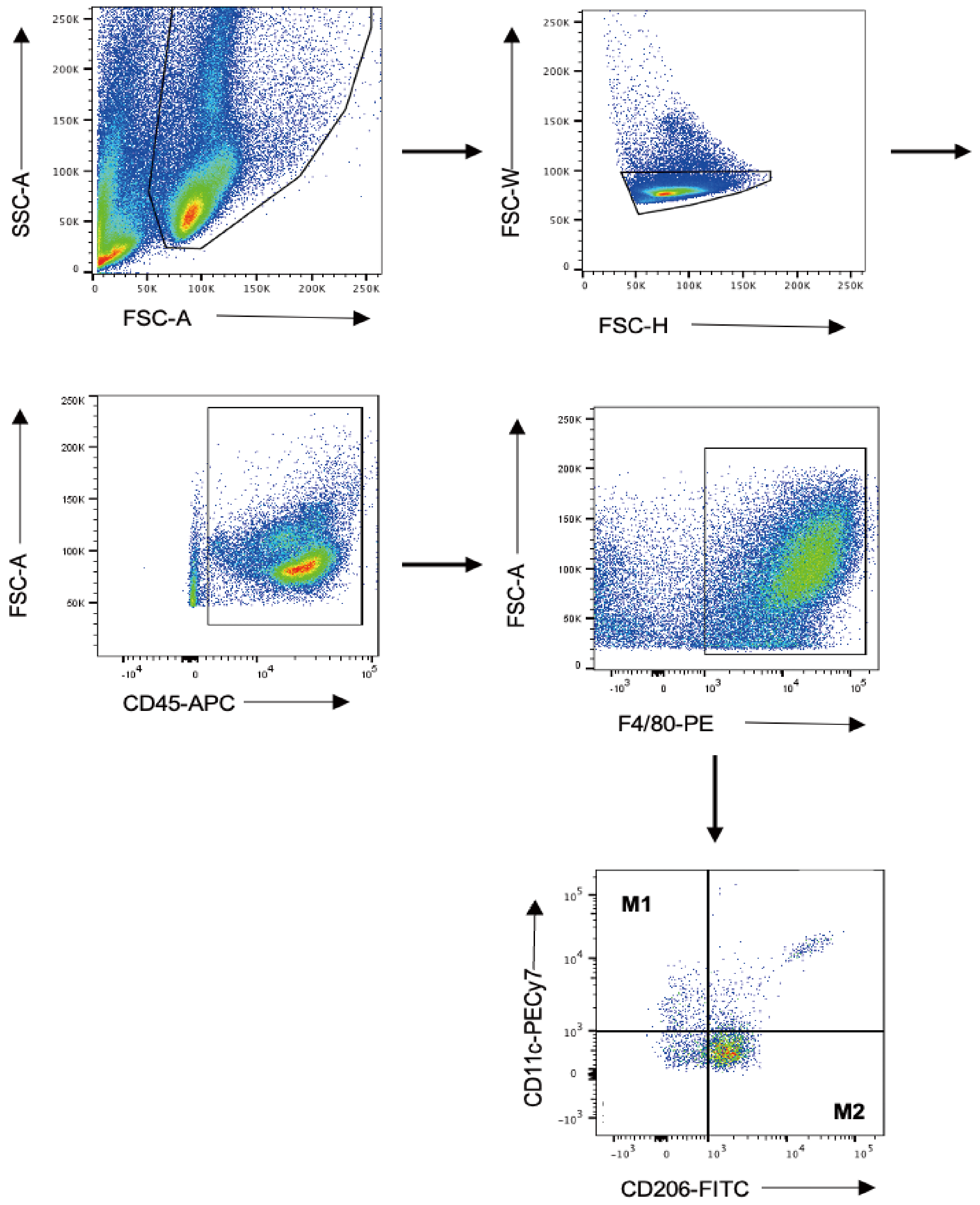

Supplementary Figure 2. Strategy for macrophages

Representative flow cytometry plots of liver CD45+ F4/80+ CD206- CD11c+ M1 macrophages and CD45+ F4/80+ CD206+ CD11c- M2 macrophages in each group at 16 weeks of age.

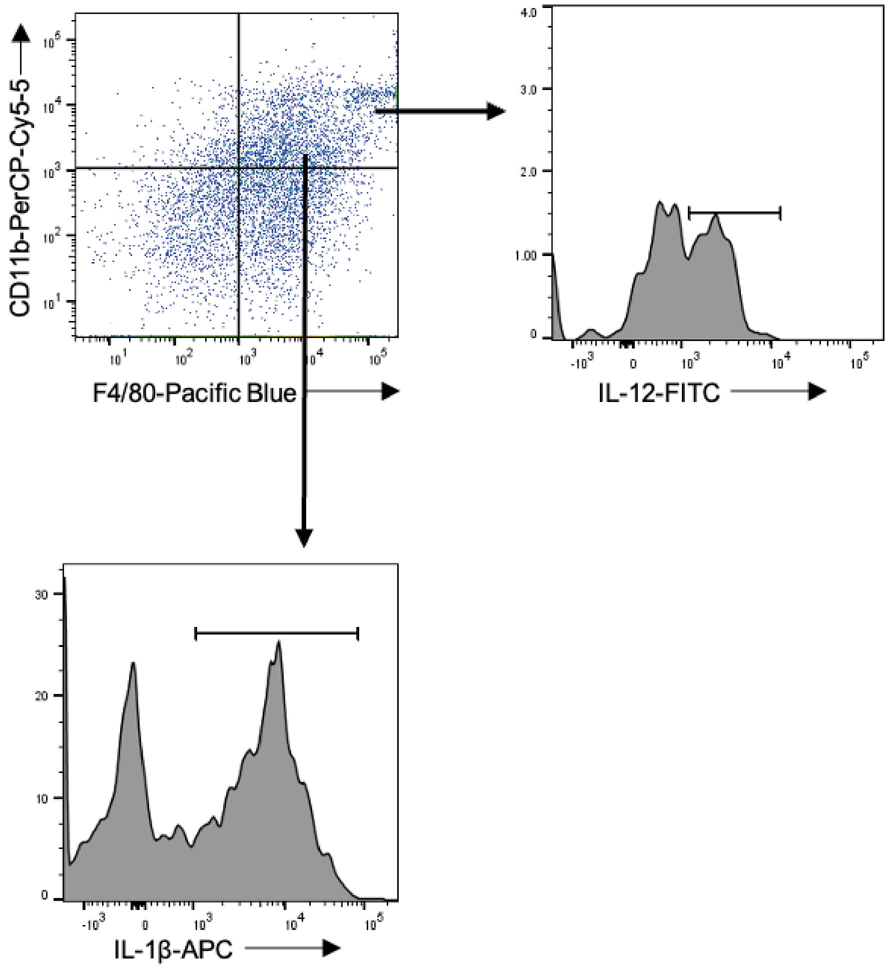

Supplementary Figure 3. Strategy for macrophages

Representative flow cytometry plots of RAW264.7 cells F4/80+ CD11b+ IL12 cells and F4/80+ CD11b+ IL1β cells.

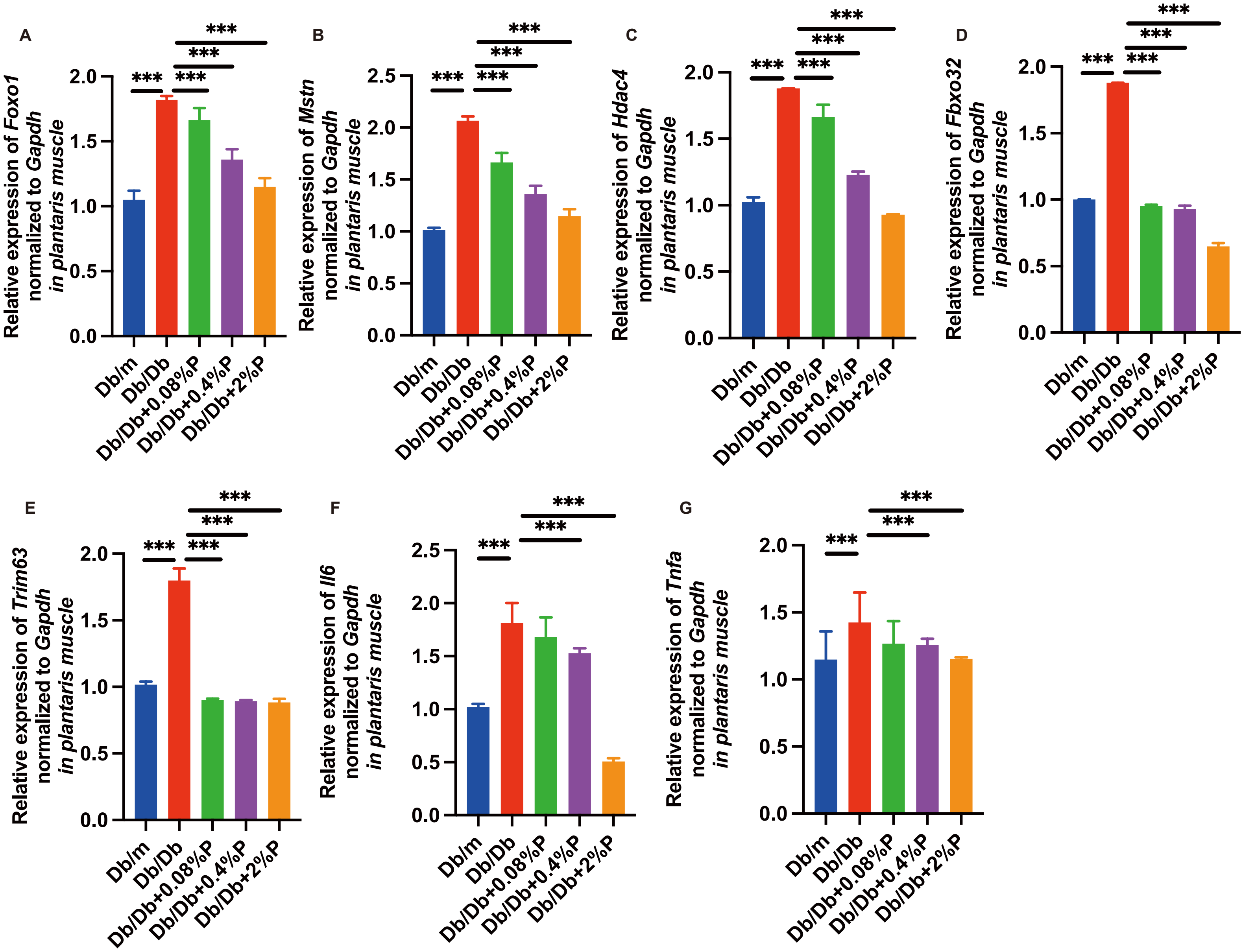

Supplementary FIgure 4. The gene expressions in plantaris muscle.

The relative expression of mRNA (A) *Foxo1*, (B) *Mstn*, (C) *Hdac4*, (D) **Fbxo32**, (E) *Trim63*, (F) *Il6*, and (G) *Tnfa* of indicated genes in soleus muscle normalized to Gapdh (n=12).

Data have been represented in terms of mean ± SD values. ***p<0.001 by one-way ANOVA.

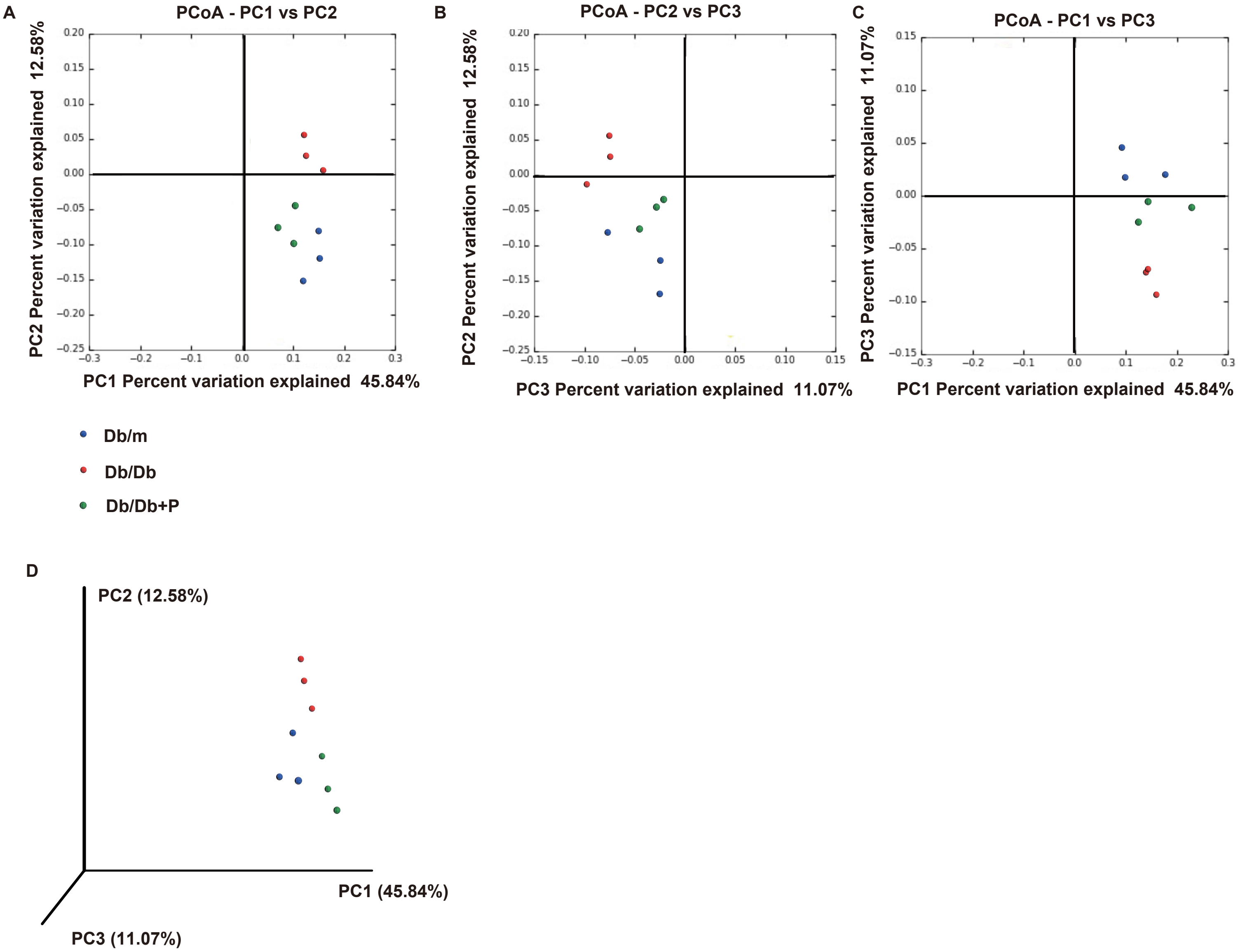

Supplementary Figure 5. PCoA plots of gut microbiota of Db/m, Db/Db, and Eb/DB+2%P mice.

Weighted PCoA plots were shown (n=3). (A) PC1 vs PC2, (B) PC2 vs PC3, and (C) PC1 vs PC3.

(D) Weighted beta-diversity with PERMANOVA test were shown (n=3).

PCoA: Principal coordinate analysis, PERMANOVA: Permutational multivariat analysis of variance.

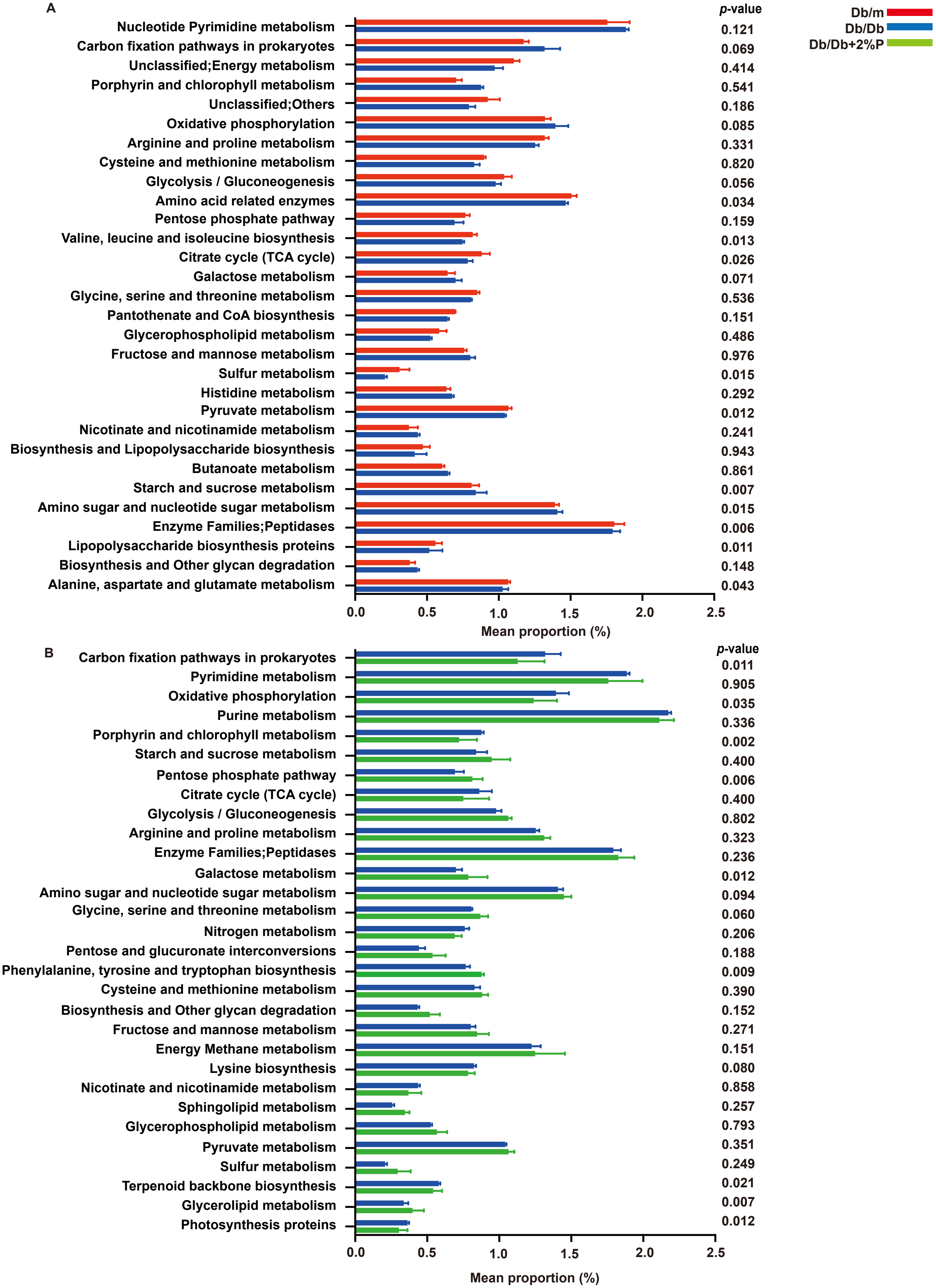

A
Db/m
Db/Db
Db/Db+2%P
p-value
Nucleotide Pyrimidine metabolism 0.121
Carbon fixation pathways in prokaryotes 0.069
Unclassified;Energy metabolism 0.414
Porphyrin and chlorophyll metabolism 0.541
Unclassified;Others 0.186
Oxidative phosphorylation 0.085
Arginine and proline metabolism 0.331
Cysteine and methionine metabolism 0.820
Glycolysis / Gluconeogenesis 0.056
Amino acid related enzymes 0.034
Pentose phosphate pathway 0.159
Valine, leucine and isoleucine biosynthesis 0.013
Citrate cycle (TCA cycle) 0.026
Galactose metabolism 0.071
Glycine, serine and threonine metabolism 0.536
Pantothenate and CoA biosynthesis 0.151
Glycerophospholipid metabolism 0.486
Fructose and mannose metabolism 0.976
Sulfur metabolism 0.015
Histidine metabolism 0.292
Pyruvate metabolism 0.012
Nicotinate and nicotinamide metabolism 0.241
Biosynthesis and Lipopolysaccharide biosynthesis 0.943
Butanoate metabolism 0.861
Starch and sucrose metabolism 0.007
Amino sugar and nucleotide sugar metabolism 0.015
Enzyme Families;Peptidases 0.006
Lipopolysaccharide biosynthesis proteins 0.011
Biosynthesis and Other glycan degradation 0.148
Alanine, aspartate and glutamate metabolism 0.043
0.0 0.5 1.0 1.5 2.0 2.5
Mean proportion (%)
B
p-value
Carbon fixation pathways in prokaryotes 0.011
Pyrimidine metabolism 0.905
Oxidative phosphorylation 0.035
Purine metabolism 0.336
Porphyrin and chlorophyll metabolism 0.002
Starch and sucrose metabolism 0.400
Pentose phosphate pathway 0.006
Citrate cycle (TCA cycle) 0.400
Glycolysis / Gluconeogenesis 0.802
Arginine and proline metabolism 0.323
Enzyme Families;Peptidases 0.236
Galactose metabolism 0.012
Amino sugar and nucleotide sugar metabolism 0.094
Glycine, serine and threonine metabolism 0.060
Nitrogen metabolism 0.206
Pentose and glucuronate interconversions 0.188
Phenylalanine, tyrosine and tryptophan biosynthesis 0.009
Cysteine and methionine metabolism 0.390
Biosynthesis and Other glycan degradation 0.152
Fructose and mannose metabolism 0.271
Energy Methane metabolism 0.151
Lysine biosynthesis 0.080
Nicotinate and nicotinamide metabolism 0.858
Sphingolipid metabolism 0.257
Glycerophospholipid metabolism 0.793
Pyruvate metabolism 0.351
Sulfur metabolism 0.249
Terpenoid backbone biosynthesis 0.021
Glycerolipid metabolism 0.007
Photosynthesis proteins 0.012
0.0 0.5 1.0 1.5 2.0 2.5
Mean proportion (%)

Supplementary FIgure 6. The functional profiles for gut microbiota.

(A and B) Change in metabolism profiles for the gut microbiota.

The degree of impairment in the gut microbial metabolism profile for Db/m mice, Db/Db mice, and Db/Db+2%P mice was assessed by the weighted average differences method, and the degree was ranked from top to bottom. The top 20 metabolism profiles for gut microbiota are shown, and the difference of these gut microbiota profiles was evaluated by unpaired t‐tests. Histograms show the mean proportion of the relative abundance of metabolism profiles of gut microbiota (mean + standard deviation) and *p*‐value by unpaired t‐test is shown.

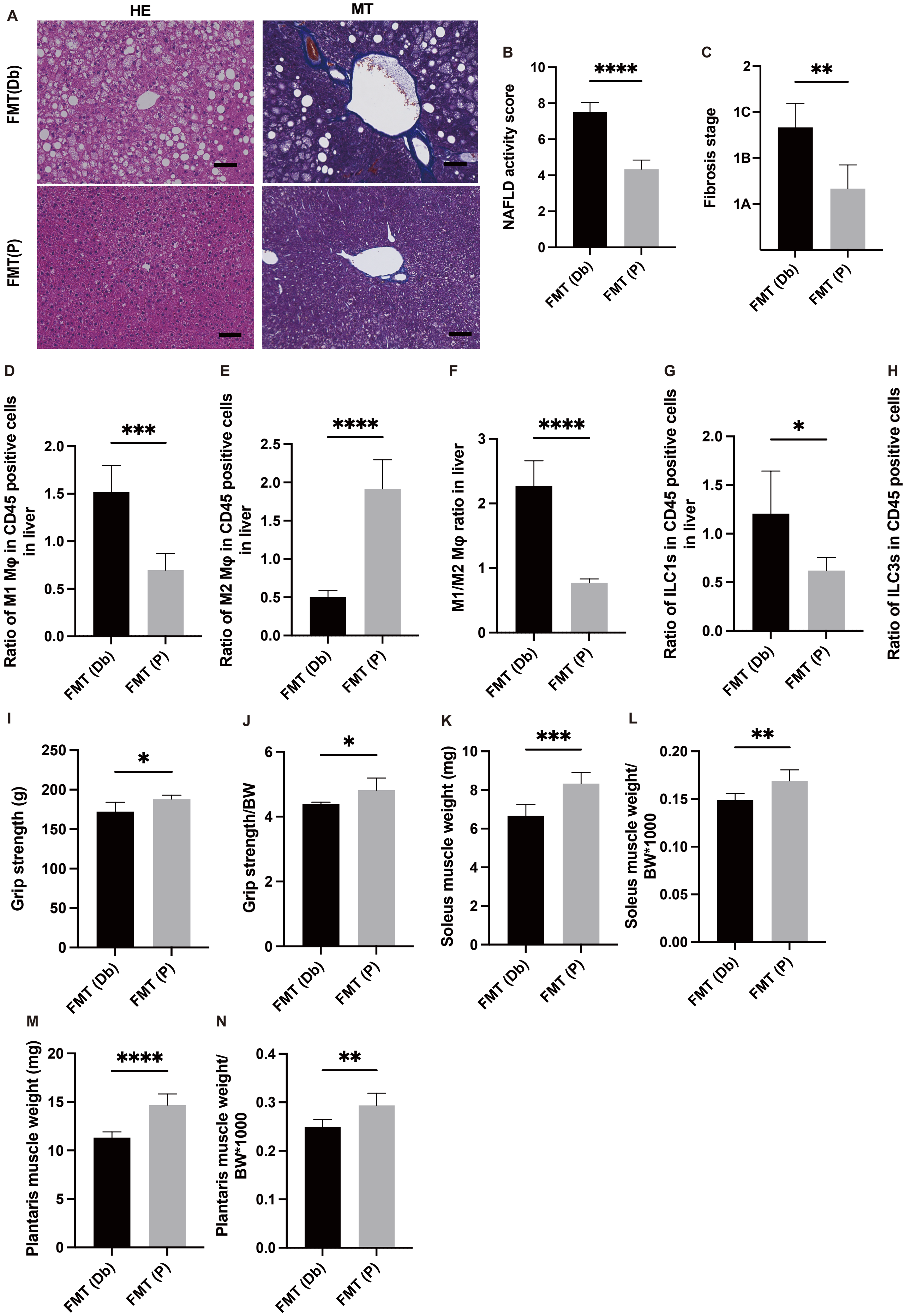
A
HE
MT
FMT(Db)
FMT(P)
B
NAFLD activity score
****
C
Fibrosis stage
**
D
Ratio of M1 Mφ in CD45 positive cells in liver
***
E
Ratio of M2 Mφ in CD45 positive cells in liver
****
F
M1/M2 Mφ ratio in liver
****
G
Ratio of ILC1s in CD45 positive cells in liver
*
H
Ratio of ILC3s in CD45 positive cells in liver
**
I
Grip strength (g)
*
J
Grip strength/BW
*
K
Soleus muscle weight (mg)
***
L
Soleus muscle weight/ BW*1000
**
M
Plantaris muscle weight (mg)
****
N
Plantaris muscle weight/ BW*1000
**
FMT (Db)
FMT (P)

Supplementary Figure 7. Fecal microbiota transplantation of Db/Db+2%P mice improved fatty liver, decreased the expression of genes related to inflammation, liver fibrosis, and fatty acid metabolism, and decreased the ratio of M1 macrophages, ILC1, and ILC3, and increased the ratio of M2 macrophages and ILC2, and increased the muscle strength and mass.

(A) Representative images of hematoxylin & eosin- and Masson trichrome-stained liver sections. Liver tissues were collected at 16-weeks of age. The scale bar shows 100 μm.

(B and C) Nonalcoholic fatty liver disease (NAFLD) activity scores and the fibrosis stage of NAFLD activity score (n = 6). Ratio of (D) M1 macrophages to CD45-positive cells, (E) M2 macrophages to CD45-positive cells, (F) M1 to M2 macrophages, (G) ILC1s to CD45-positive cells, (H) ILC3s to CD45-positive cells in the liver (n = 6 in each case). (I and J) Absolute and relative grip strength, (K and L) absolute and relative soleus muscle weight, (M and N) absolute and relative plantaris muscle weight in 16-weeks-old mice (n = 12 in each case).

Data are represented as the mean ± SD values. Data were analyzed using unpaired t-test.

*$p<0.05$, **$p<0.01$, ***$p<0.001$.

FMT, fecal microbiota transplantation.
